# Saharan dust induces NLRP3-dependent inflammatory cytokines in an alveolar air-liquid interface co-culture model

**DOI:** 10.1186/s12989-023-00550-w

**Published:** 2023-10-20

**Authors:** Gerrit Bredeck, Jochen Dobner, Burkhard Stahlmecke, Khanneh Wadinga Fomba, Hartmut Herrmann, Andrea Rossi, Roel P. F. Schins

**Affiliations:** 1 IUF – Leibniz Research Institute for Environmental Medicine, 40225 Düsseldorf, Germany; 2 Institut für Umwelt & Energie, Technik & Analytik e. V. (IUTA), 47229 Duisburg, Germany; 3 Atmospheric Chemistry Department (ACD), Leibniz Institute for Tropospheric Research (TROPOS), 04318 Leipzig, Germany

**Keywords:** African dust, Lung inflammation, Alveolar epithelium, In vitro, Endotoxin

## Abstract

**Background:**

Epidemiological studies have related desert dust events to increased respiratory morbidity and mortality. Although the Sahara is the largest source of desert dust, Saharan dust (SD) has been barely examined in toxicological studies. Here, we aimed to assess the NLRP3 inflammasome-caspase-1-pathway-dependent pro-inflammatory potency of SD in comparison to crystalline silica (DQ12 quartz) in an advanced air-liquid interface (ALI) co-culture model. Therefore, we exposed ALI co-cultures of alveolar epithelial A549 cells and macrophage-like differentiated THP-1 cells to 10, 21, and 31 µg/cm² SD and DQ12 for 24 h using a Vitrocell Cloud system. Additionally, we exposed ALI co-cultures containing *caspase* (*CASP*)*1*^−/−^ and *NLRP3*^−/−^ THP-1 cells to SD.

**Results:**

Characterization of nebulized DQ12 and SD revealed that over 90% of agglomerates of both dusts were smaller than 2.5 μm. Characterization of the ALI co-culture model revealed that it produced surfactant protein C and that THP-1 cells remained viable at the ALI. Moreover, wild type, *CASP1*^−/−^, and *NLRP3*^−/−^ THP-1 cells had comparable levels of the surface receptors cluster of differentiation 14 (CD14), toll-like receptor 2 (TLR2), and TLR4. Exposing ALI co-cultures to non-cytotoxic doses of DQ12 and SD did not induce oxidative stress marker gene expression. SD but not DQ12 upregulated gene expressions of interleukin 1 Beta (*IL1B*), *IL6*, and *IL8* as well as releases of IL-1β, IL-6, IL-8, and tumor necrosis factor α (TNFα). Exposing wild type, *CASP1*^−/−^, and *NLRP3*^−/−^ co-cultures to SD induced *IL1B* gene expression in all co-cultures whereas IL-1β release was only induced in wild type co-cultures. In *CASP1*^−/−^ and *NLRP3*^−/−^ co-cultures, IL-6, IL-8, and TNFα releases were also reduced.

**Conclusions:**

Since surfactants can decrease the toxicity of poorly soluble particles, the higher potency of SD than DQ12 in this surfactant-producing ALI model emphasizes the importance of readily soluble SD components such as microbial compounds. The higher potency of SD than DQ12 also renders SD a potential alternative particulate positive control for studies addressing acute inflammatory effects. The high pro-inflammatory potency depending on NLRP3, CASP-1, and IL-1β suggests that SD causes acute lung injury which may explain desert dust event-related increased respiratory morbidity and mortality.

**Supplementary Information:**

The online version contains supplementary material available at 10.1186/s12989-023-00550-w.

## Background

Epidemiological studies have revealed that desert dust exposure increases respiratory morbidity and mortality [1–5]. In toxicological studies, desert dusts from different regions have been demonstrated to induce oxidative stress and inflammatory signaling. Especially Asian sand dust [6, 7], Middle East desert dust [8, 9], and Northern American desert dust [10, 11] have been tested in animal models as well as in cell culture models of epithelial cells and macrophages. Studies comparing desert dusts from different sources or different dust events reported variations in toxic potencies related to their heterogeneous composition [7, 9, 12, 13]. Desert dusts are mixtures containing amongst others inorganic particles, metals, and ions as well as organic carbon compounds, microbial components such as fungal β-glucans and bacterial endotoxins, and even viable microbes [7, 9, 14, 15].

Although Saharan dust (SD) accounts for over 50% of the global desert dust emission [16], few studies have addressed its toxicity: Organic extracts from SD collected in Puerto Rico [17–19] and SD containing urban pollutants from Mali [20] were shown to trigger oxidative stress and inflammation using in vitro models of bronchial epithelial cells. Furthermore, we could recently demonstrate that SD collected on the Cape Verde islands induced oxidative stress and inflammatory cytokines in alveolar epithelial A549 cells as well as pro-inflammatory cytokine secretion from macrophage-like THP-1 cells [21]. The latter was strongly dependent on the NACHT, LRR, and PYD domains-containing protein 3 (NLRP3) inflammasome.

Activation of the NLRP3 inflammasome is a two-step process that leads to the secretion of the pro-inflammatory cytokine interleukin (IL)-1β from innate immune cells such as macrophages. The first, or priming, step can be mediated by toll-like receptor (TLR) signaling which is triggered by microbial danger signals, e.g. lipopolysaccharides [22, 23]. Upon priming the expressions of NLRP3 and pro-IL-1β are upregulated [22, 23]. The second, or activation, step can be mediated by multiple stimuli, including bacterial pathogens and their components, and particles such as quartz (extensively reviewed in [24]). In the activation step, the NLRP3 inflammasome is assembled, leading to the activation of caspase (CASP)-1, which in turn cleaves pro-IL-1β to mature IL-1β [25, 26]. Mature IL-1β induces further cytokines such as IL-6 and IL-8 [27–29]. The NLRP3-mediated production of IL-1β has a critical role in acute lung injury [30–32] and is crucial in the development of particle-induced lung inflammation and fibrosis, i.e. silicosis [33, 34]. Apart from IL-1β, the NLRP3 inflammasome-CASP-1 pathway also leads to the release of IL-18 from macrophages [33, 35]. The role of IL-18 in silicosis is less well established than of IL-1β [36–38]. Besides IL-1β, tumor necrosis factor α (TNFα) is the other critical early pro-inflammatory cytokine for the development of acute lung injury [39] and silicosis [40].

In this study, we analyzed the effects of SD in an air-liquid interface (ALI) co-culture model of A549 and differentiated THP-1 cells combining two major advantages. Firstly, the culture at the ALI enables more realistic exposure conditions and causes more realistic properties, e.g. production of surfactant and a lower surface tension through higher amounts of lipids [41–43]. Secondly, the co-culture of both cell types enables their cross-talk. Cytokines released from macrophages can substantially enhance the cytokine response of epithelial cells [44, 45]. The other way around, contact with epithelial cells shapes the activity of alveolar macrophages [46–49]. For instance, surfactant proteins A and C have been reported to attenuate the activations of TLR2 [48], TLR4 [49], and cluster of differentiation (CD)14 [50] through microbial components. Moreover, the surfactant lining can facilitate the uptake of particles by macrophages and epithelial cells [51]. Also, surfactants can decrease the cytotoxicity towards alveolar macrophages and the pro-inflammatory activity of quartz dust [52–54]. While desert dust represents one of the main sources of global non-anthropogenic air pollution, to the best of our knowledge, to date, desert dust has neither been tested in a co-culture of epithelial cells and macrophages nor in an ALI co-culture model.

Using this advanced ALI co-culture model, we aimed to compare the potencies of SD and DQ12 quartz dust to induce oxidative and pro-inflammatory signaling. In addition, we sought to analyze the role of the NLRP3 inflammasome regarding potential pro-inflammatory effects in this ALI co-culture model.

## Results

### Size distribution of nebulized dusts

**Fig. 1:**
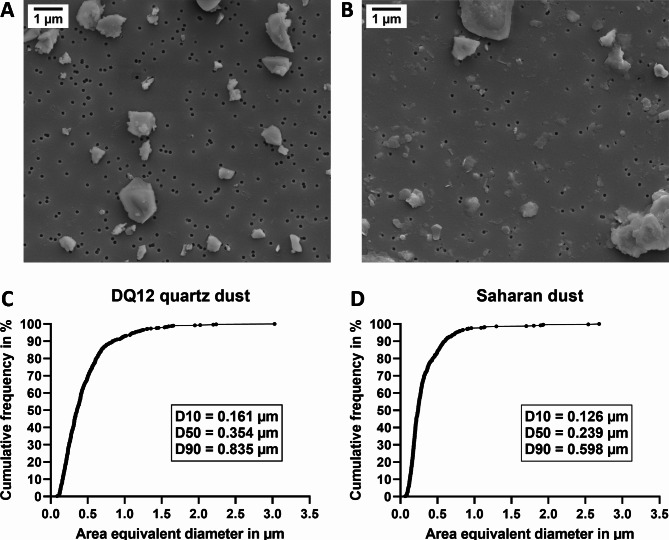
**Cumulative agglomerate size distributions of area equivalent diameters.** Suspensions of DQ12 quartz dust (**A**, **C**) and Saharan dust (**B**, **D**) in endotoxin-free H_2_O containing 1.25% PBS were sonicated and nebulized onto 0.1 μm pore-size nucleopore filters. SEM images were obtained at a nominal magnification of 5,000 x (pixel size: 6.2 nm). Images **A** and **B** show excerpts from images used for size determination. For 500 agglomerates, the area equivalent diameter was determined based on Feret_max_ and Feret_min_ diameters (**C**, **D**)

To assess the size distribution of DQ12 and SD deposited on the ALI co-cultures, we examined nebulized dust by scanning electron microscopy (SEM) (Fig. 1; Fig. S1, Additional File 1). Since a particle can consist of smaller (primary) particles that agglomerated to a bigger compact particle which is considered to be relevant for exposure, we did not assess the primary particle size distribution but the agglomerate size distribution. The sizes of agglomerates (including single particles) were log-normally distributed with mode diameters of 223 nm (variance of σ = 2.18) and 176 nm (variance of σ = 1.55) for DQ12 and SD, respectively. Nebulized DQ12 consisted of larger agglomerates than SD. About 93% and 97% of DQ12 and SD agglomerates were smaller than 1.0 μm, respectively. More than 99% of agglomerates of both dusts were smaller than 2.5 μm.

### Characterization of the in vitro model

**Fig. 2:**
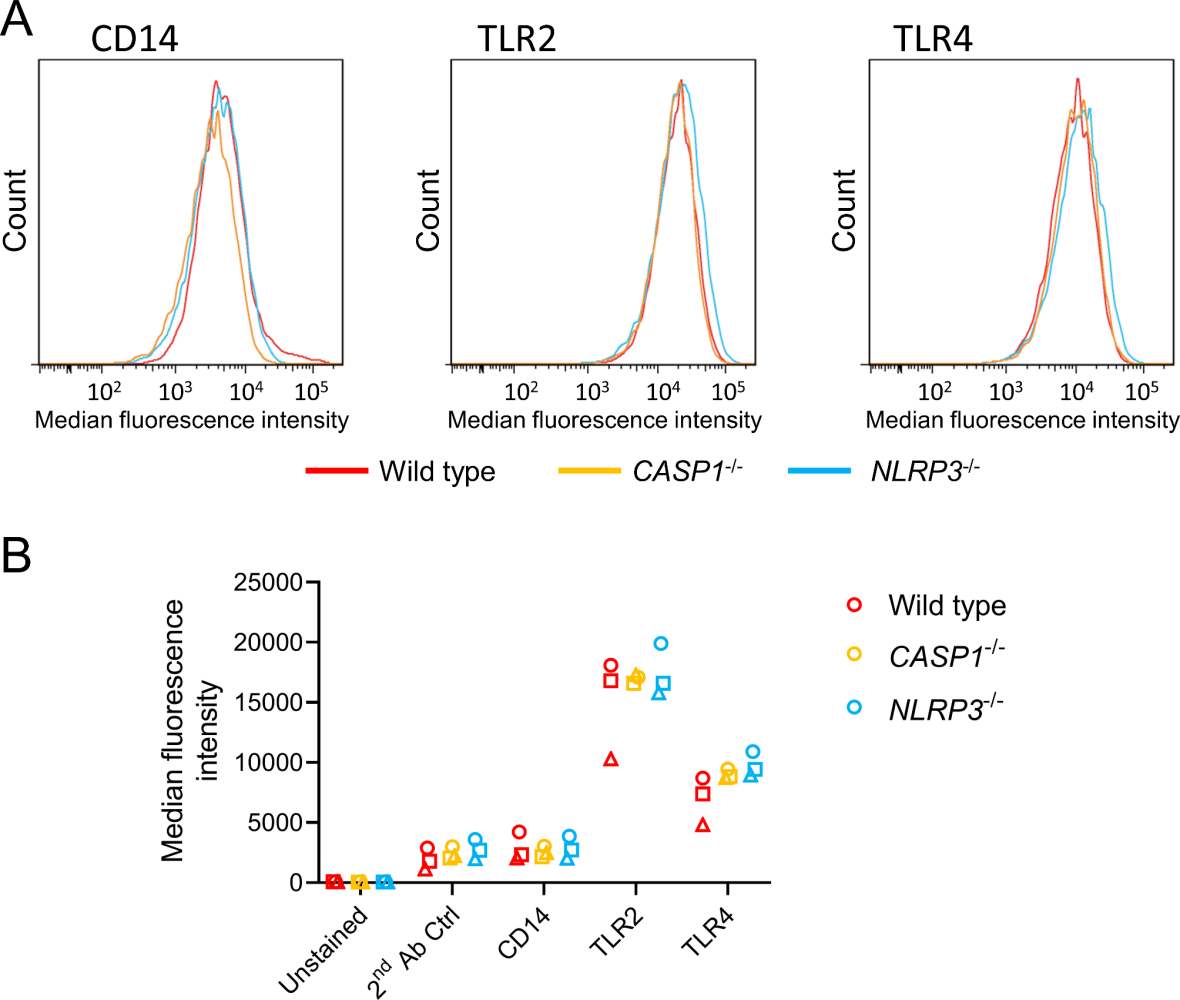
**Surface receptor levels of CD14, TLR2, and TLR4 of differentiated THP-1 cells.** Differentiated wild type, *CASP1*^−/−^, and *NLRP3*^−/−^ THP-1 cells were immuno-stained for surface CD14, TLR2, or TLR4. Fluorescence intensities were measured by flow cytometry. Representative histograms are shown in **A**. The median fluorescence intensities from *N* = 3 independent experiments are plotted in **B** (one biological replicate per group). A two-way ANOVA with Tukey’s post hoc test was calculated (no significant differences between genotypes obtained; different shapes represent experimental runs)

To analyze whether the surface levels of the receptors CD14, TLR2, and TLR4 were different between differentiated wild type, *CASP1*^−/−^, and *NLRP3*^−/−^ THP-1 cells, we performed immuno-staining and assessed the cells via flow cytometry (Fig. 2; Fig. S2, Additional File 2). We did not observe significant differences in surface levels between the genotypes for any of the investigated receptors. These results indicate that wild type, *CASP1*^−/−^, and *NLRP3*^−/−^ THP-1 cells can be used to assess the role of the NLRP3 inflammasome-CASP-1 pathway without confounding through differential surface levels of upstream receptors.

**Fig. 3:**
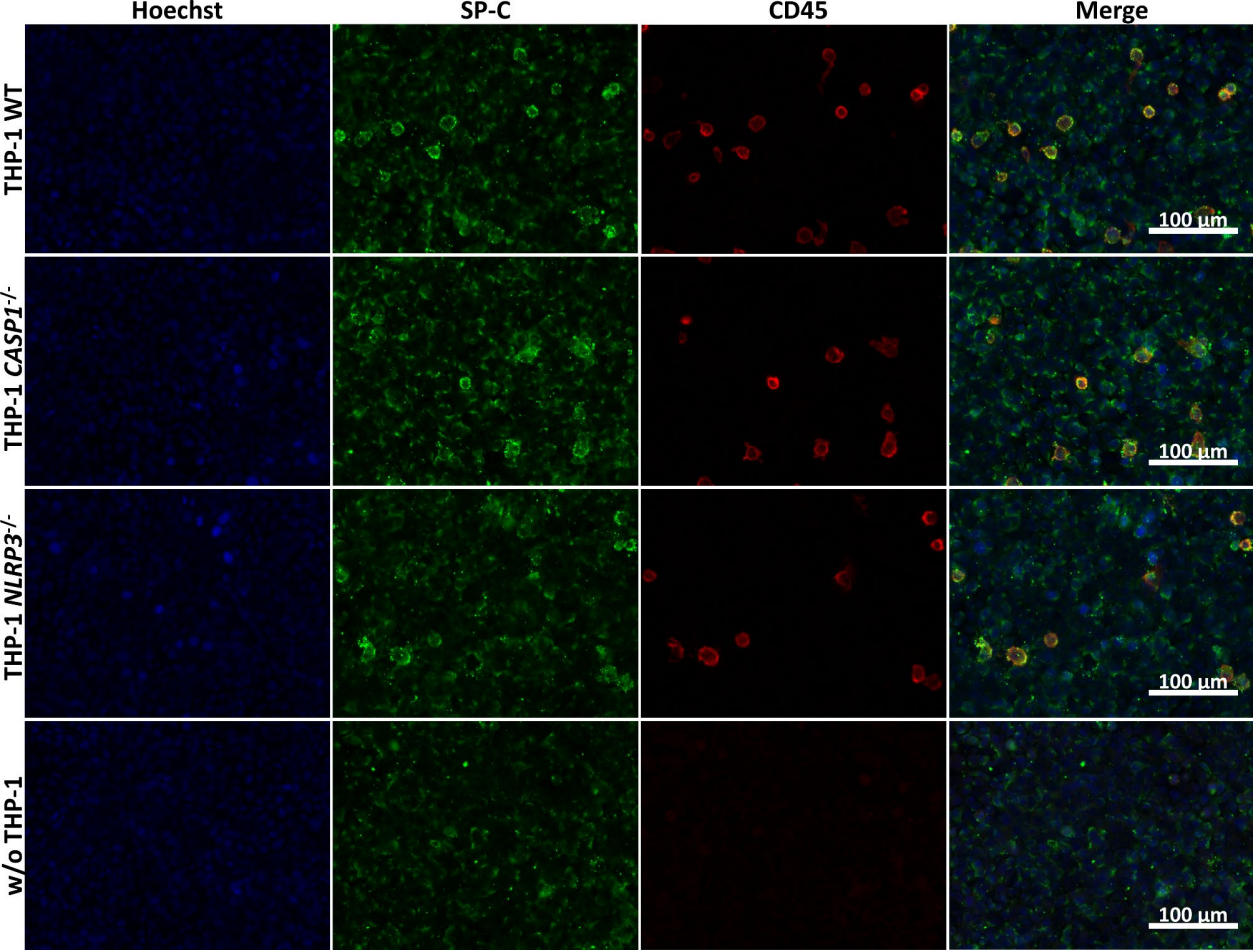
**Surfactant production and macrophage viability at the air-liquid interface (ALI).** Co-cultures of A549 cells with wild type (WT), *CASP1*^−/−^, and *NLRP3*^−/−^ THP-1 cells as well as A549 mono-cultures were fixed after 48 h of cultivation at the ALI. Nuclei were stained with Hoechst 33342. Immunostaining of surfactant protein C (SP-C) and CD45 was performed. Representative images were obtained at 100 x magnification

We analyzed the capacity of co-cultures of A549 cells with wild type, *CASP1*^−/−^, and *NLRP3*^−/−^ THP-1 cells and A549 mono-cultures to produce surfactant protein C (SP-C) by immuno-staining and visualization by fluorescence microscopy (Fig. 3). Simultaneously, we examined the presence of differentiated THP-1 cells after 48 h of ALI culture via staining of CD45. We observed slightly stronger staining of SP-C for the co-cultures than for A549 mono-cultures. The genotype of the THP-1 cells did not influence the intensity of SP-C staining. CD45 staining indicated that THP-1 cells of all genotypes were still present after 48 h of ALI culture. The correct genotypes of THP-1 cells were confirmed by submerged lipopolysaccharide (LPS) exposure of THP-1 cell mono-cultures in parallel to ALI co-culture experiments and comparison of the IL-1β release to the results from a previous study (Fig. S3, Additional File 3) [55].

### Toxicities of DQ12 quartz dust and Saharan dust at the air-liquid interface

We exposed the co-cultures to DQ12 and SD at the ALI, using a Vitrocell Cloud 12α. To verify exposure to the dusts and visualize the morphology of the nebulized dusts, transmission electron microscopy (TEM) grids were loaded in parallel to the ALI co-cultures and were subsequently assessed via SEM (Fig. S4, Additional File 4). Due to surface tension, the dusts on the TEM grids agglomerated during drying. The agglomerates of both dusts were of a rather compact shape. DQ12 appeared to have sharper edges than SD. The deposited doses were quantified via quartz crystal microbalance (sQCM) (Table S1, Additional File 5).

**Fig. 4:**
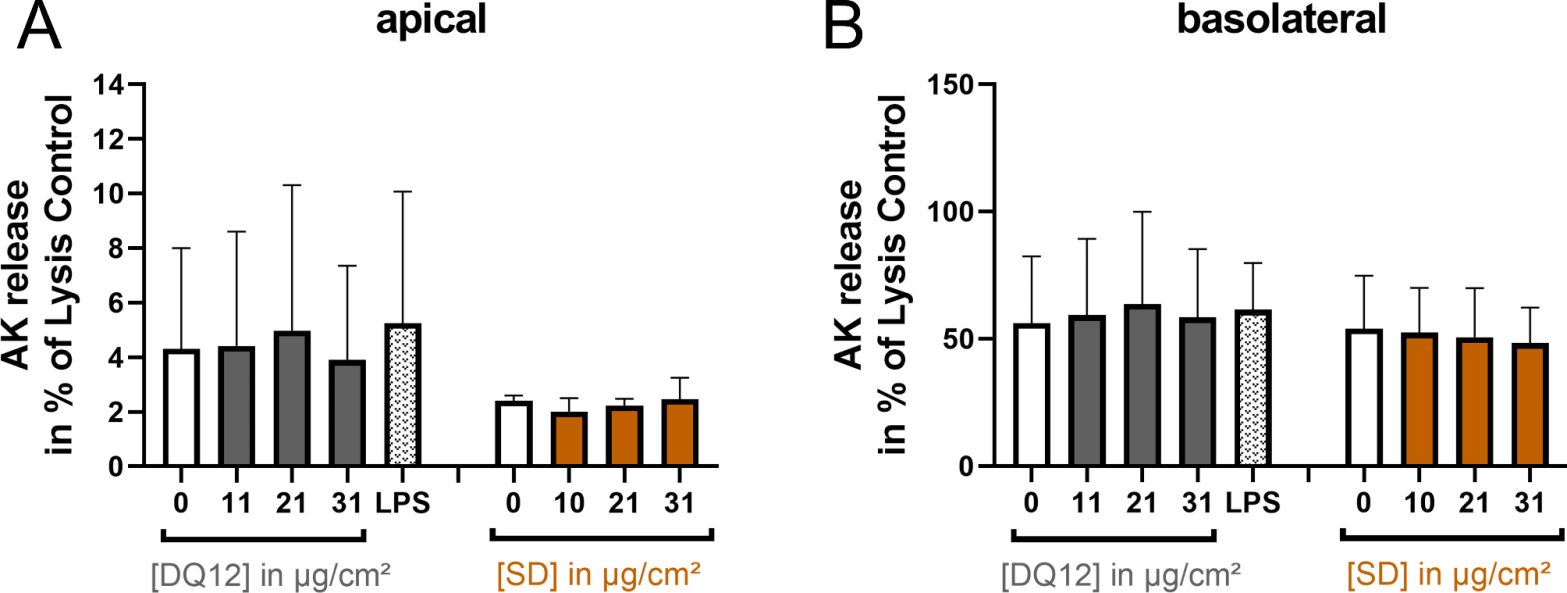
**DQ12 quartz dust and Saharan dust were not cytotoxic**. Air-liquid interface (ALI) co-cultures were exposed to DQ12 and Saharan dust (SD) at doses in the range of 0–31 µg/cm² for 24 h or to 250 ± 60 ng/cm² LPS for 21 h. The apical (**A**) and basolateral (**B**) release of adenylate kinase (AK) was examined as a marker of cytotoxicity using the ToxiLight assay. ALI cultures treated with 0.5% Triton X-100 served as lysis control. The values are plotted as means ± standard deviation of *N* = 4 independent experiments (Controls and LPS: one or two biological replicates; DQ12 and SD; duplicates per concentration). A mixed-effects model with Šidák’s post hoc test was calculated (no significant differences obtained)

**Absence of cytotoxicity.** To monitor the cytotoxicity of DQ12, SD, and LPS in the ALI co-cultures, we examined the release of adenylate kinase (AK) (Fig. 4). Neither DQ12 nor SD induced cytotoxicity at the tested doses. The release of AK into the basolateral compartment was relatively high, compared to the lysis control. This could be due to the short incubation time of 15 min with Triton X-100 which was not sufficient to allow diffusion of AK to the basolateral compartment.

**Fig. 5:**
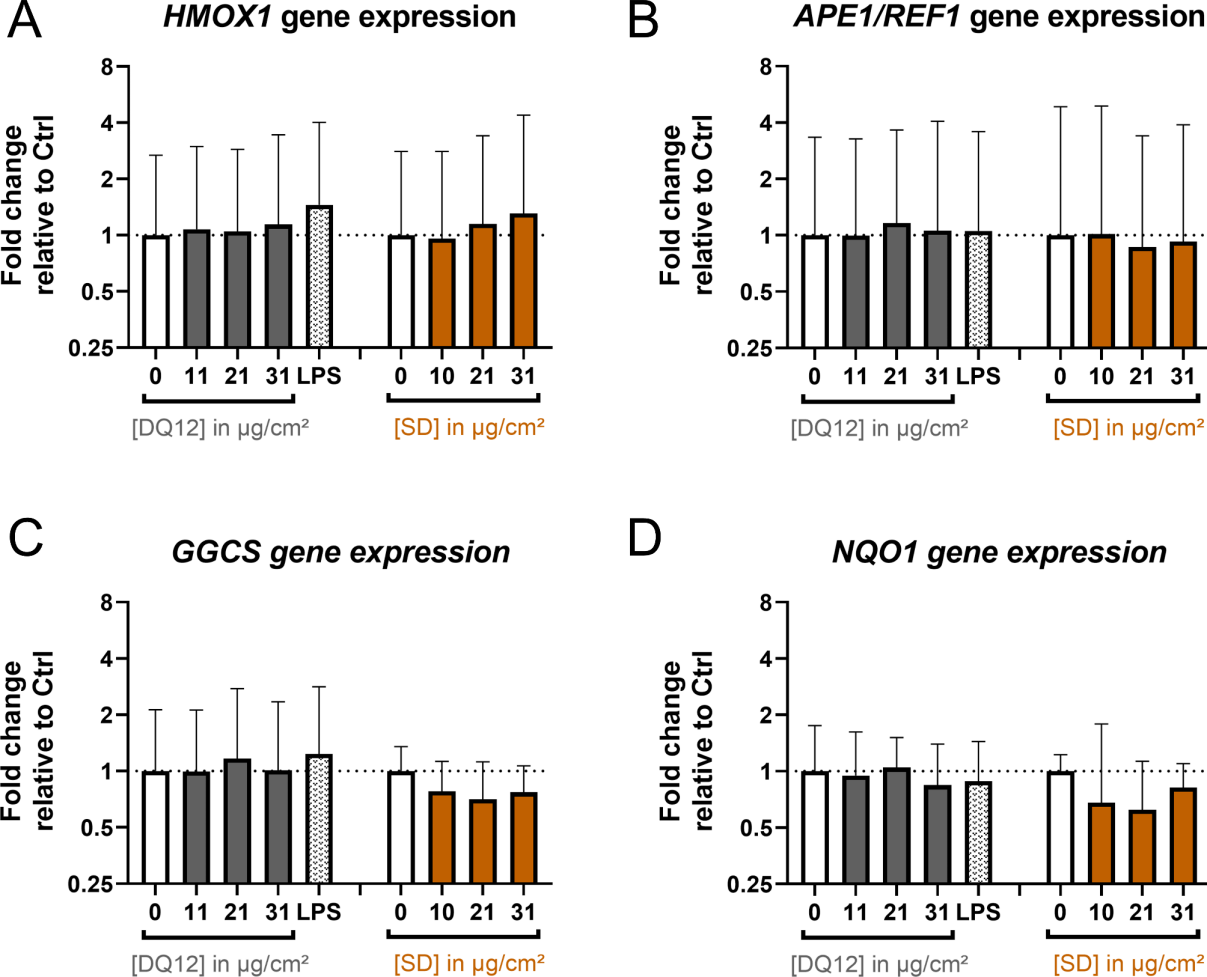
**DQ12 quartz dust and Saharan dust did not induce oxidative stress marker gene expression.** Air-liquid interface co-cultures were exposed to DQ12 and Saharan dust (SD) at doses in the range of 0–31 µg/cm² for 24 h or 250 ± 60 ng/cm² LPS for 21 h. The relative gene expression of *HMOX1* (**A**), *APE1/REF1* (**B**), *GGCS* (**C**), and *NQO1* (**D**) was assessed by qRT-PCR. The results were normalized to control ALI cultures as well as to the reference genes *ACTB* and *GAPDH*. The depicted fold changes with standard deviations were derived from the means of the ΔΔC_T_ values and standard deviations of the ΔC_T_ values of *N* = 4 independent experiments (Controls and LPS: one or two biological replicates; DQ12 and SD: duplicates per concentration). A mixed-effects model with Šidák’s post hoc test was calculated based on the ΔC_T_ values (no significant differences obtained)

**Absence of oxidative stress.** To analyze oxidative stress, we assessed the mRNA levels of the marker genes heme oxygenase 1 (*HMOX1*), apurinic-apyrimidinic endonuclease 1/redox factor 1 (*APE1/REF1)*, gammaglutamylcysteine synthetase (*GGCS*), and NAD(P)H quinone dehydrogenase (*NQO1*) via quantitative reverse transcription polymerase chain reaction (qRT-PCR) (Fig. 5). None of the investigated marker genes was dysregulated upon 24 h of exposure to DQ12 or SD. The positive control for inflammatory effects, LPS, was tested in parallel. LPS did not induce the oxidative stress marker genes either.

**Fig. 6:**
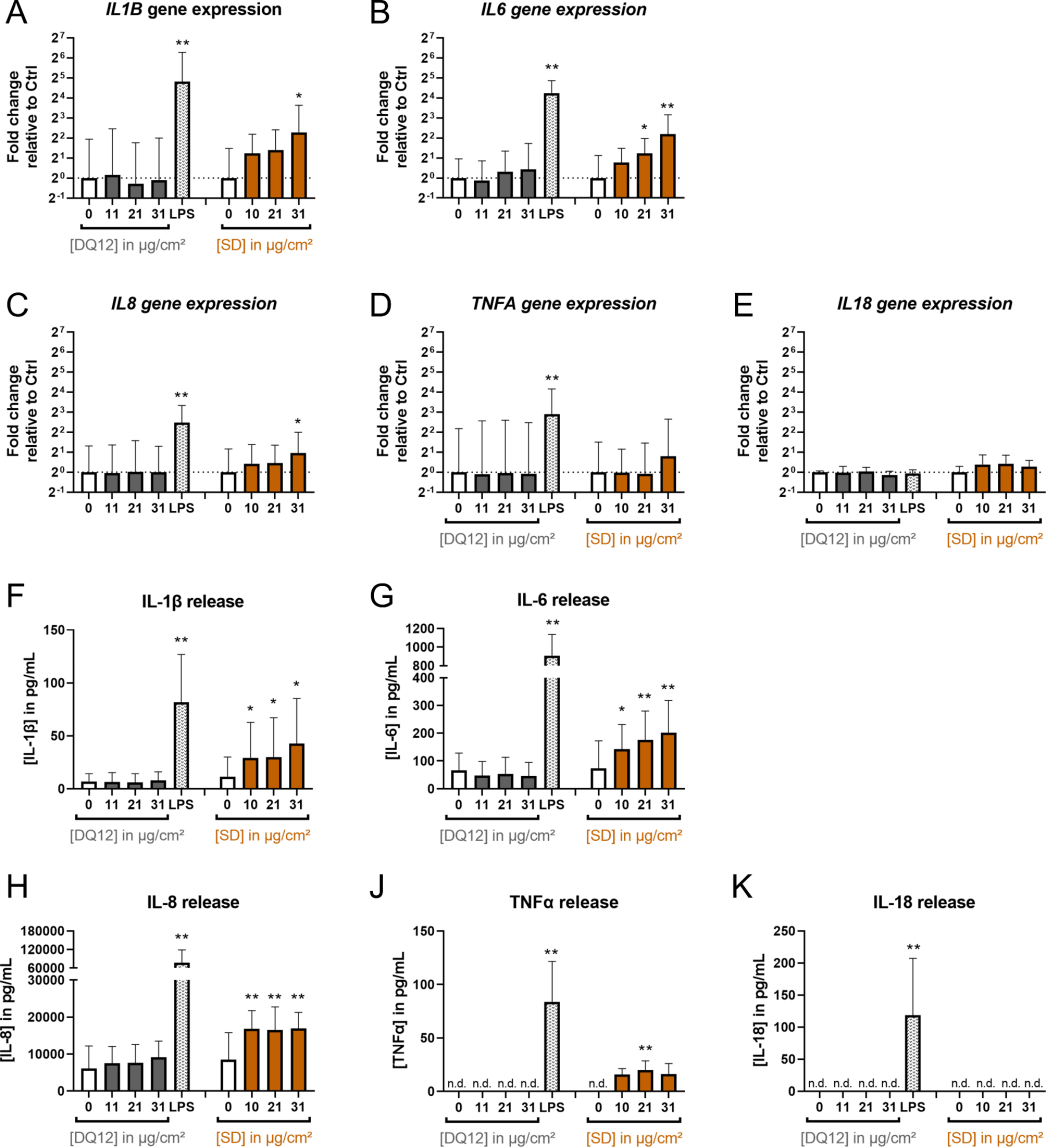
**Saharan dust but not DQ12 quartz dust induced cytokine gene expression and release.** Air-liquid interface co-cultures were exposed to DQ12 and Saharan dust (SD) at doses in the range of 0–31 µg/cm² for 24 h or 250 ± 60 ng/cm² LPS for 21 h. The relative gene expressions of *IL1B* (**A**), *IL6* (**B**), *IL8* (**C**), *TNFA* (**D**), and *IL18* (**E**) as well as basolateral cytokine releases of IL-1β (**F**), IL-6 (**G**), IL-8 (**H**), TNFα (**J**), and IL-18 (**K**) were assessed by qRT-PCR and ELISA, respectively. The gene expression results were normalized to control ALI cultures as well as to the reference genes *ACTB* and *GAPDH*. Fold changes were derived from the means of ΔΔC_T_ values and standard deviations of ΔC_T_ values. Depicted are mean values with standard deviations. Mixed-effects models with Šidák’s post hoc test were calculated based on the ΔC_T_ values and absolute cytokine concentrations for qRT-PCR and ELISA data, respectively. *N* = 4 independent experiments were performed (*N* = 3 for IL-18 release; Controls and LPS: one or two biological replicates; DQ12 and SD: duplicates per concentration; *p ≤ 0.05; **p ≤ 0.01). n.d.: not detected

**Pro-inflammatory effects of SD.** To analyze pro-inflammatory effects, we examined the gene expressions of *IL1B*, *IL6*, *IL8*, *TNFA*, and *IL18* and cytokine releases of IL-1β, IL-6, IL-8, TNFα, and IL-18 (Fig. 6). Surprisingly, DQ12 did not affect any of these cytokines. On the other hand, SD significantly enhanced cytokine gene expression and release. The highest tested dose of 31 µg/cm² SD upregulated *IL1B* and *IL6* gene expression as well as IL-1β and IL-6 release about three to five-fold. At the same dose, *IL8* gene expression and IL-8 release were upregulated about two-fold. *TNFA* gene expression was not significantly upregulated. TNFα release was induced at all tested SD doses whereas only at the medium dose statistical significance was reached. *IL18* gene expression was not affected by SD exposure. The concentrations of released IL-18 remained below the limit of detection for controls and all SD doses. The increases in the expressions of *IL1B*, *IL6*, and *IL8* as well as release of IL-1β and IL-6 showed a dose-dependent trend. The lack of this trend for IL-8 release might be due to increasing interference with the IL-8 ELISA with increasing SD dose (see Fig. S5, Additional File 6). As expected, the positive control LPS strongly induced the gene expression of *IL1B*, *IL6*, *IL8*, and *TNFA* and the release of all investigated cytokines. LPS did not affect the gene expression of *IL18*. In summary, SD was a more potent inducer of pro-inflammatory cytokines than DQ12.

**Fig. 7:**
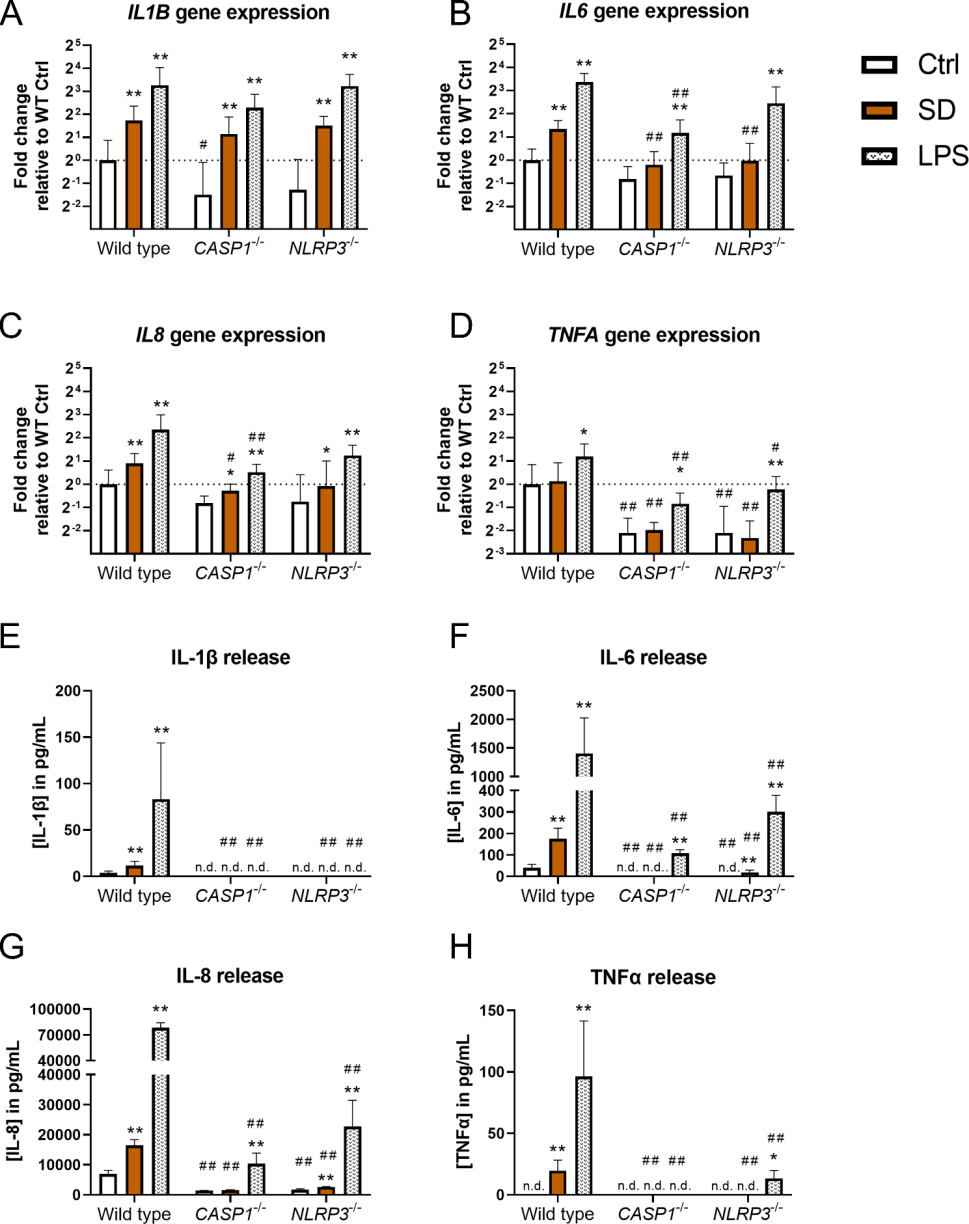
**Caspase-1 and NLRP3 dependent cytokine gene expression and release in response to Saharan dust.** Air-liquid interface co-cultures with either wild type, *caspase (CASP)1*^−/−^, or *NLRP3*^−/−^ THP-1 cells were exposed to 31 µg/cm² SD for 24 h or to 260 ± 30 ng/cm² LPS for 21 h. The relative gene expressions of *IL1B* (**A**), *IL6* (**B**), *IL8* (**C**), and *TNFA* (**D**) as well as basolateral cytokine releases of IL-1β (**E**), IL-6 (**F**), IL-8 (**G**), and TNFα (**H**) were assessed by qRT-PCR and ELISA, respectively. The gene expression results were normalized to wild type control ALI cultures as well as to the reference genes *ACTB* and *GAPDH*. Fold changes were derived from the means of ΔΔC_T_ values and standard deviations of ΔC_T_ values. Depicted are mean values with standard deviations. Mixed-effects models with Šidák’s post hoc test were calculated based on the ΔC_T_ values and absolute cytokine concentrations for qRT-PCR and ELISA data, respectively. *N* = 4 independent experiments were performed (Control and LPS: one biological replicate per genotype; SD: duplicates; Comparisons to the non-exposed controls of the respective genotype: *p ≤ 0.05; **p ≤ 0.01; Comparisons to equally exposed wild type co-cultures: ^#^*p* ≤ 0.05; ^##^*p* ≤ 0.01) n.d.: not detected

**NLRP3 inflammasome dependence of cytokine releases.** To address the role of the NLRP3 inflammasome, we exposed ALI co-cultures with THP-1 wild type and CRISPR/Cas9-generated THP-1 *CASP1*^−/−^ and *NLRP3*^−/−^ cells (hereinafter “wild type co-cultures”, “*CASP1*^−/−^ co-cultures”, and “*NLRP3*^−/−^ co-cultures”) to 31 µg/cm² SD or the positive control LPS (see sQCM-measured doses in Table S2, Additional File 7). SD upregulated *IL1B* gene expression irrespective of the THP-1 cells’ genotype (Fig. 7). SD exposure only induced the release of IL-1β from wild type co-cultures. In contrast, for *CASP1*^−/−^ and *NLRP3*^−/−^ co-cultures, no IL-1β could be detected in the basolateral supernatant, not even after stimulation with LPS.

As in wild-type co-cultures, SD exposure enhanced the gene expression of *IL6* in *CASP1*^−/−^ and *NLRP3*^−/−^ co-cultures, though statistical significance was not reached (*p* = 0.067 and *p* = 0.052, respectively). The IL-6 concentration in the basolateral supernatants from SD-exposed *CASP1*^−/−^ co-cultures was below the limit of detection, whereas augmented IL-6 release from SD-exposed *NLRP3*^−/−^ co-cultures was detected.

SD exposure upregulated the gene expression of *IL8* by about 1.5 to two-fold irrespective of the genotype. We observed an increase of IL-8 release in wild type and *NLRP3*^−/−^ but not in *CASP1*^−/−^ co-cultures. Yet, interference of SD with the IL-8 ELISA could have masked an upregulation in *CASP1*^−/−^ co-cultures (see Fig. S5, Additional File 6).

SD exposure did not lead to enhanced gene expression of *TNFA* for any of the investigated genotypes. TNFα release was only stimulated in wild type co-cultures, while the TNFα concentrations were below the limit of detection for SD-exposed *CASP1*^−/−^ and *NLRP3*^−/−^ co-cultures.

For all genotypes, LPS exposure strongly induced the gene expressions and releases of *IL6*/IL-6, *IL8*/IL-8, and *TNFA*/TNFα. Within each exposure group, the released concentrations of IL-6, IL-8, and TNFα from the co-cultures followed the order wild type > *NLRP3*^−/−^ > *CASP1*^−/−^. We confirmed the correct genotypes of THP-1 cells by submerged LPS exposure of THP-1 cell mono-cultures in parallel to ALI co-culture experiments and comparison of the IL-1β release to the results from a previous study (Fig. S3, Additional File 3) [55].

In summary, deficiencies of CASP-1 and NLRP3 abrogated the SD-induced secretion of IL-1β. In addition, deficiencies of CASP-1 and NLRP3 reduced the gene expressions and releases of *IL6*/IL-6, *IL8*/IL-8, and *TNFA*/TNFα across all exposure groups.

## Discussion

In this study, we tested Saharan dust (SD) toxicity in an advanced ALI co-culture model. This model produces surfactant and includes the crosstalk between epithelial and macrophage-like cells. In our exposure system, the SD deposited on the ALI co-cultures was in a respirable size range. In the ALI co-culture model, SD had a higher pro-inflammatory potency than DQ12 quartz dust (DQ12). SD but not DQ12 induced IL-1β, IL-6, IL-8, and TNFα. Further, our data show that the NLRP3 inflammasome-CASP-1 pathway is essential to boost the inflammatory response to SD exposure. In *CASP1*^−/−^ and *NLRP3*^−/−^ co-cultures, SD did not trigger IL-1β secretion, and the releases of IL-6, IL-8, and TNFα were substantially lower than in wild type co-cultures.

### Advantages of the air-liquid interface co-culture model

Here, we applied an advanced in vitro ALI co-culture model comprising alveolar epithelial A549 cells and macrophage-like differentiated THP-1 cells. This enabled us to test desert dust toxicity in a more advanced model than traditional submerged monocultures. The ALI co-culture model produces surfactant which can modify the toxicity of particles [51, 56, 57] and microbial compounds [50, 58, 59]. The ALI co-culture model also includes the important crosstalk between epithelial cells and macrophages [44–49]. Furthermore, the ALI co-culture model allows us to do mechanistic research by using specific knock-out models. Here, we could specifically address the role of the NLRP3 inflammasome-CASP-1 pathway in macrophages. We could confirm by flow cytometry that the deficiencies of NLRP3 and CASP-1 did not affect the levels of relevant surface receptors upstream of NLRP3 inflammasome activation, i.e., CD14, TLR2, and TLR4 [22, 60]. In addition, this ALI co-culture model offers the opportunity to selectively introduce further genetic modifications to epithelial cells or macrophages. Therefore, this in vitro model could be used instead of conditional mutant animals.

Using the ALI co-culture model, we could test SD in comparison to well-investigated DQ12 quartz dust. In our Vitrocell Cloud exposure system, the size range of the deposited agglomerates matches the respirable particle size range [61]. The doses tested in our study correspond to concentrations of quartz dust that were necessary to cause oxidative stress and inflammatory cytokine upregulation in previous in vitro studies by us and others [62–64]. Testing of SD concentrations that are at least as high as quartz concentrations is supported by the expected doses in acute exposure scenarios: Quartz dust is an occupational pollutant and maximum air concentrations are regulated. In contrast, concentrations of the environmental pollutant SD cannot be regulated. During dust events, SD concentrations can even exceed the maximum occupational concentrations of respirable quartz of 50 µg/m³ and 100 µg/m³ in the USA [65] and the European Union [66], respectively [67, 68].

So far, alveolar epithelial ALI co-culture models have especially been applied to toxicity testing of nanomaterials [69–71]. Moreover, such models have been used to assess environmental pollutants [72, 73] and crystalline silica [62, 71]. Here, to the best of our knowledge, for the first time, an ALI co-culture model is applied to assess desert dust toxicity. Overall, the use of this advanced in vitro model for the assessment of the toxicity of SD agrees with the replacement of animal studies as an essential part of the 3 Rs by Russell and Burch [74], particularly considering the high animal numbers required for in vivo knock-out models.

### Higher acute pro-inflammatory potency of Saharan dust than DQ12 quartz dust

Notably, in this advanced ALI co-culture model, SD but not DQ12 induced the gene expression and release of pro-inflammatory cytokines. Because of its known potential to trigger NLRP3-dependent cytokine production, DQ12 had originally been included as a positive control [34]. The higher inflammatory potency of SD compared to DQ12 contrasts existing studies which compared desert and quartz dusts. These contrasts may be explained by different potencies amongst different desert dusts and by the use of different models. The pro-inflammatory potency of SD widely agrees with the existing literature on desert dust toxicity both in in vivo [6, 8, 11, 75–77] and in vitro [10, 12, 17, 19, 21] studies.

Contrasting our results, Ghio et al. [10] found pro-inflammatory cytokine releases which were similar or stronger upon quartz dust exposure compared to desert dust exposure. They exposed mice and human bronchial epithelial BEAS-2B cells to Arizona surface dust and quartz dust. Similarly, Kim et al. [78] exposed rat lung alveolar epithelial RLE-6TN cells to Asian yellow sand dust and quartz dust and observed comparable potencies to induce TNFα secretion. A possible explanation for the different inflammatory potencies between the desert dust sample from our study and other studies may be the different sampling locations [9, 10, 13]. In particular, the microbial composition could be the factor that distinguishes our Saharan dust sample and dust samples from other deserts, considering that even at the same sampling location there can already be substantial daily variability in microbial composition [79]. Furthermore, as we reported previously [21], our Saharan dust sample contains iron, aluminum, trace metals, sulfate, diatomaceous earth, and organic and elemental carbon; constituents that are absent or barely contained in quartz dust, that can vary between desert dust samples and that potentially contribute to toxicity.

Contrasts between our and other studies may also be due to varying toxic potencies in different in vitro models. For instance, in the in vitro models of the aforementioned studies by Ghio et al. [10] and Kim et al. [78] no macrophages were included. Macrophages could potentiate the pro-inflammatory cytokine response to microbial components that are contained in SD but not DQ12 [21]. Additionally, differences between in vitro models are reflected by the huge range of quartz dust concentrations needed in different studies to obtain inflammatory effects in vitro, reaching from 0.23 µg/cm² [80] to 320 µg/cm² [81]. The use of different models must also explain the higher inflammatory potency of SD than DQ12 and the low inflammatory potency of DQ12 in the present study compared to our previous study [21]. In the previous study, we tested the same SD and DQ12 samples in submerged mono-cultures of A549 and differentiated THP-1 cells: In A549 cells, DQ12 induced *IL8* expression even more potently than SD after 24 h of exposure. In differentiated TPH-1 cells, SD and DQ12 induced IL-1β release with similarly strong potencies. The higher relative potency of SD compared to DQ12 may be explained by the surfactant produced by the ALI co-culture model. Surfactant can decrease the toxicity of poorly soluble particles [52–54]. The stronger effects of poorly soluble particles in submerged than ALI co-culture models are in line with studies directly comparing the sensitivities of submerged versus ALI models [62, 73, 82]. Accordingly, in our more realistic ALI co-culture model, readily soluble components such as microbial compounds that are only contained in SD but not in DQ12 would become more important and drive SD-triggered inflammatory signaling.

In the ALI co-culture model, we did not observe oxidative stress in response to SD or DQ12 although oxidative stress after desert dust exposure [10, 21, 83] and quartz dust exposure [21, 63, 84] was reported in previous studies using submerged models. This difference between submerged models and the ALI co-culture model may again be explained by the lower potency of poorly soluble particles in ALI models. Moreover, the absence of oxidative stress in our study suggests that the inflammatory potency of SD is oxidative stress independent. This agrees with our previous study of SD in submerged A549 cells. In the A549 cells, SD upregulated *IL6* and *IL8* expression at 4 h but not at 24 h whereas oxidative stress was only induced at the later time point [21].

In contrast to acute effects, persistent inflammatory effects could not be addressed using our ALI co-culture model since at extended cultivation times the cells overgrow. Still, due to the failure of clearance, persistent inflammation, which can lead to fibrosis, is especially relevant for poorly soluble particles such as quartz dust [85, 86]. In studies on camels, desert dust exposure has been correlated with persistent inflammation and lung fibrosis as well [87, 88]. To investigate these potential chronic effects of desert dust in vitro, more sophisticated models will be required such as primary cell tissues or organoids.

### NLRP3 inflammasome and caspase-1 dependence

Our results suggest that SD can mediate the priming step of the NLRP3 inflammasome-CASP-1 pathway and that SD-triggered IL-1β release strongly depends on the NLRP3 inflammasome and CASP-1. The transcriptional upregulation of *IL1B* gene expression suggests SD-induced priming of the NLRP3 inflammasome-CASP-1 pathway. Especially, the upregulation of *IL1B* gene expression in *CASP1*^−/−^ and *NLRP3*^−/−^ co-cultures supports the ability of SD to induce the priming step as in these co-cultures *IL1B* gene expression cannot have been upregulated by autocrine IL-1β signaling. In concordance with our results, He et al. [6] found increased expression of *NLRP3* and *IL1B* in Asian sand dust (ASD)-exposed murine macrophage-like cells. That desert dust can prime the NLRP3 inflammasome-CASP-1 pathway is not surprising, considering its content of microbial components [79]. The dependence of SD-induced IL-1β secretion on the NLRP3 inflammasome-CASP-1 pathway agrees with the findings from our previous study of SD in wild type and *NLRP3*^−/−^ THP-1 mono-cultures [21]. Also, we found that SD did not induce the NLRP3 inflammasome-CASP-1 pathway-dependent cytokine IL-18. The difference between IL-1β and IL-18 induction aligns well with a study by Midtbö et al. [89]. Their study also agrees with our findings regarding the incapacity of LPS to induce *IL18* gene expression in THP-1 cells. Which components of SD drive the specificity for IL-1β over IL-18 induction remains to be determined.

Additionally, our results show that beyond the direct downstream cytokine IL-1β, the functional NLRP3 inflammasome boosts the secretion of the pro-inflammatory cytokines IL-6, IL-8, and TNFα upon SD exposure. The dependence of IL-8 secretion on IL-1β concentrations has also been described by other authors [44, 45]. For the inductions of IL-6 and IL-8 secretion, we found the same dependences on exposure and genotype. IL-6 and IL-8 have in common that the NFκB pathway contributes to their regulation [27, 28]. Further supporting the boost of secondary cytokines through the NLRP3 inflammasome-CASP-1 pathway, CASP-1 has been demonstrated to activate the transcription factor NF-κB via caspase-7 activation, so without the necessity of IL-1β activation [90]. Furthermore, IL-1β has been suggested to induce TNFα via the NFκB pathway, though the exact mechanism is still being discussed [91]. Further studies are required to unravel the involvement of the NFκB pathway in the inflammatory effects of SD.

Besides NLRP3, further inflammasomes may play a role in SD-induced inflammatory signaling, as well. The involvement of further inflammasomes could explain the higher IL-6 and IL-8 secretion from *NLRP3*^−/−^ co-cultures than from *CASP1*^−/−^ co-cultures. Amongst others, the NLRP1 [92], NLRC4 (or IPAF) [93], AIM2 [94], and NLRP6 (or PYPAF5) [95] inflammasomes have been reported to activate caspase-1. The potential dependence of SD toxicity on the listed inflammasomes is emphasized by the fact that they can be induced by different microbial components (reviewed by [96, 97]) and that the bacterial endotoxin LPS also had stronger effects on *NLRP3*^−/−^ co-cultures than on *CASP1*^−/−^ co-cultures. Our ALI co-culture model is suitable to address the role of these inflammasomes in future studies using similar genetic-targeting approaches as used in the current study.

## Conclusion

In conclusion, in an advanced air-liquid interface co-culture model, only Saharan dust but not DQ12 quartz dust upregulated pro-inflammatory cytokines. As discussed in our previous study, a main difference between Saharan and quartz dust is the presence of readily soluble components, especially microbial compounds [21]. Here we suggest that in the more realistic ALI co-culture model, the soluble components are more relevant for toxicity. Which soluble and especially which microbial compounds contribute to Saharan dust’s toxicity needs to be addressed in further studies.

Moreover, considering the higher potency of Saharan dust and the absence of quartz dust toxicity which has also been observed in other studies [98, 99], we suggest using Saharan dust as an alternative particulate positive control. A well-characterized and stored batch of Saharan dust could be used in studies addressing acute inflammatory effects.

Furthermore, the pro-inflammatory effects of Saharan dust in the absence of quartz dust-induced effects and the association with the NLRP3-caspase-1-IL-1β axis strongly underline its hazardousness. To understand the roles of the NLRP3 inflammasome-caspase-1 pathway’s priming and activation step more closely, the expression of NLRP3 and the cleavage of CASP-1 should be addressed in further studies. Considering the importance of the NLRP3 inflammasome in acute lung injury [30–32], it could mechanistically link desert dust events to the epidemiologically observed increases in respiratory morbidity and mortality [1–5]. In future studies, the involvement of the NLRP3 inflammasome-caspase-1 pathway could be addressed by analyzing the susceptibility towards desert dust-mediated morbidities depending on single nucleotide polymorphisms in the NLRP3 gene. In addition to the NLRP3 inflammasome-caspase-1 pathway, further mechanisms must be involved in desert dust toxicity as indicated by the residual Saharan dust-induced pro-inflammatory effects in *CASP1*^−/−^ and *NLRP3*^−/−^ co-culture. Given the stronger effects in *NLRP3*^−/−^ than in *CASP1*^−/−^ co-cultures, other inflammasomes should be assessed. Future studies should unravel these mechanisms.

## Materials and methods

### Chemicals and reagents

RPMI-1640 media, 2-mercaptoethanol (ME), Hank’s Balanced Salt Solution containing MgCl_2_ and CaCl_2_ (HBSS), sodium pyruvate, and TRIzol® reagent were purchased from Thermo Fisher Scientific. Fetal calf serum (FCS), bovine serum albumin (BSA), Penicillin/Streptomycin (P/S), D-glucose, trypsin, accutase, phorbol 12-myristate-13-acetate (PMA), LPS, endotoxin-free H_2_O, phosphate-buffered saline (PBS), 4-(2-hydroxyethyl)-1-piperazineethanesulfonic acid (HEPES), 2-propanol, and the amplification grade DNase I Kit were purchased from Sigma-Aldrich/Merck. The ToxiLight assay kit was purchased from Lonza. Ethanol and sulfuric acid were purchased from Roth. Nuclease-free water was purchased from Qiagen. The iScript™ cDNA Synthesis Kit and the iQ™ SYBR® Green Supermix were purchased from Bio-Rad. Primers for qPCR were purchased from Eurofins. The Human IL-1β, IL-6, IL-8, and TNFα DuoSet ELISA Kits were purchased from R&D Systems.

### Dusts

The collection and characterization of the SD sample have been described previously [21]. Dörentrup Quartz (DQ12) was used. To ensure DQ12 was free of biological contaminants, it was baked at 220 °C overnight before application to cells.

### Scanning electron microscopy

To assess the agglomerate size distribution applied to the co-cultures, dusts were suspended to 4 mg/mL in endotoxin-free H_2_O containing 1.25% PBS. The suspensions were probe sonicated at 6.9 W for 17 min to reach a total delivered acoustic energy of 7056 J using a Branson Sonifier 450 at 20% amplitude. A nucleopore filter (0.1 μm pore size) was placed on the moistened frit of a vacuum filtration unit and a vacuum was applied. Above the nucleopore filter, 75 µL of dust suspension was nebulized using a Vitrocell 9–12 μm nebulizer to which a 600 Hz square signal was applied using a Velleman PCSU200 Oscilloscope. Due to the vacuum, the liquid was drawn through the filter pores leaving the agglomerates on top of the filter without altering their sizes through the surface tension, which would lead to further agglomeration during drying. A layer of 5 nm gold was applied onto the filters by sputter coating (Quantum Design Q150V ES Plus) to render them conductive for SEM imaging. SEM images were obtained at a nominal magnification of 5.000 x (pixel size of 6.2 nm) and the Feret_max_ and Feret_min_ diameters of 500 particles/agglomerates were assessed using ImageJ (version 1.51f, https://imagej.nih.gov/ij). The area equivalent diameter of the particles/agglomerates was then calculated based on the Feret diameters.

To assess agglomerate deposition and morphology during ALI co-culture exposure, TEM grids were loaded with dusts during ALI exposure of co-cultures and assessed via SEM. Different from the filter samples no gold coating was applied since the TEM grids are conductive. Images of the TEM grids were obtained at different magnifications, starting with an overview of the complete grids up to high-resolution images of single agglomerates.

### Generation of *CASP1*^−/−^ and *NLRP3*^−/−^ THP-1 cells

*CASP1*^−/−^ and *NLRP3*^−/−^ THP-1 cells were generated as previously described [100]. Briefly, gRNAs were designed using the CRISPR design tool CHOPCHOP (http://chopchop.cbu.uib.no/) and cloned into a modified PX458 plasmid (Addgene #48138). The resulting bicistronic vector encoded the respective gRNA, Cas9 nuclease, and a GFP selection marker. gRNA efficiency was assessed using High-Resolution Melt Analysis (HRMA). A gRNA targeting *CASP1* exon 5 (5’-TAATGAGAGCAAGACGTGTG-3’) and *NLRP3* exon 2 (5’-GCTAATGATCGACTTCAATG-3’) was chosen for further experiments (HRMA primers, *CASP1*: Fwd 5’-CACCGTAATGAGAGCAAGACGTGTG-3’, Rev 5’-AAACCACACGTCTTGCTCTCATTAC-3’; *NLRP3*: Fwd 5’-CAGACCATGTGGATCTAGCC-3’, Rev 5’-TGTTGATCGCAGCGAAGAT-3’). THP-1 cells were electroporated using a Neon transfection system (Thermo Fisher Scientific) according to the manufacturer’s instructions. After 48 h, cells were FACS sorted and plated as single cells into 96-well plates. Cells were duplicated into maintenance and lysis plates after a week. Clones were then lysed with proteinase K and genotyped by PCR followed by deep sequencing using a miSeq Illumina sequencer and a V2 Nano cassette.

### Cell culture procedure

We worked with the cell lines A549 and THP-1 that are widely used in particle toxicology [21, 41, 42, 45, 62, 64, 70–72, 81, 82, 98]. A549 (ATCC) cells were stored in liquid nitrogen at passage nine and cultured in RPMI-1640 (containing L-glutamine) substituted with 10% FCS and 1% P/S, hereinafter A549 medium. Cells were detached with trypsin for subcultivation upon reaching about 80% confluence every third or fourth day and reseeded in 75 cm² flasks (Greiner) at densities of 5*10^5^ or 2.5*10^5^ cells, respectively. A549 cells were used at passages 2–20 after thawing. THP-1 (ATCC) cells were stored in liquid nitrogen at passage nine and cultured in RPMI-1640 (containing L-glutamine and 25 mM HEPES) substituted with 10% FCS, 1% P/S, 1 mM sodium pyruvate, 0.7% D-glucose and 50 nM ME, hereinafter THP-1 medium. THP-1 cells were maintained at densities between 2*10^5^ and 8*10^5^ cells/ml. THP-1 cells were used at passages 5–15 after thawing. Cell cultures were incubated at 37 °C, 5% CO_2_, and saturated humidity. The absence of mycoplasma was confirmed using the InvivoGen MycoStrip™ assay according to the manufacturer’s instructions.

For co-culture preparation, on day 1, 1.12*10^5^ A549 cells in 0.5 mL A549 medium, corresponding to 1*10^5^ cells/cm², were seeded on the apical side of a transwell filter (Corning, #3460) (Fig. S6, Additional File 8). To the basolateral side, 1.0 mL A549 medium was added. On day 2, the apical and basolateral A549 medium was changed. In parallel, 3*10^6^ THP-1 cells at passages 5–15 after thawing were differentiated with 100 nM PMA in 25 cm² flasks (Greiner) for 24 h. On day 3, the basolateral A549 medium was changed and the apical A549 was removed. The differentiated THP-1 cells were detached with accutase. Immediately after removing the apical A549 medium, 4.4*10^4^ differentiated THP-1 cells in 0.5 mL THP-1 medium without ME were seeded on the apical side of the confluent A549 cell layer. After attachment for 2 h, the apical THP-1 medium was removed and the co-cultures were lifted to the ALI. The co-culture was maintained in contact with air for 22–26 h until exposure on day 4. On day 5, after 24 h of exposure, supernatants and cells were harvested. The cell numbers were chosen to obtain a ratio of about 10:1 of A549 cells to differentiated THP-1 cells when starting the co-cultivation of both cell lines [62, 70, 73].

When wild type, *CASP1*^−/−^, and *NLRP3*^−/−^ THP-1 cells were used for ALI experiments, THP-1 cells were additionally exposed to LPS under submerged conditions to confirm the genotype via IL-1β ELISA. A total of 1.2*10^5^ THP-1 cells/well in 1.0 mL THP-1 medium without ME was seeded into 24-well plates (Greiner). After attachment for 1 h, the medium was removed and cells were exposed to 0 ng/mL and 10 ng/mL LPS in THP-1 medium without ME containing 1% FCS. After exposure for 24 h, the supernatants were collected.

### Flow cytometry

For analysis of macrophage-specific basal surface receptor levels, differentiated THP-1 wild type, *CASP1*^−/−^, and *NLRP3*^−/−^ cells were harvested, placed on ice, washed once each with cold PBS and FACS buffer (PBS containing 2 mM EDTA and 0.5% FCS), and resuspended in cold FACS buffer at 1 × 10^5^ cells per staining condition. Cells were then individually stained with antibodies for CD14 (1:1000, Thermo Fisher Scientific, #14-0149-82), TLR2 (1:250, Thermo Fisher Scientific, #14-9922-82), or TLR4 (1:500, Thermo Fisher Scientific, #14-9917-82) for 25 min on ice in the dark. Unstained and secondary antibody-only controls were each resuspended in cold FACS buffer. Afterwards, cells were washed twice with cold FACS buffer and incubated with an anti-mouse Alexa647-conjugated donkey-anti-mouse (1:250; Dianova) secondary antibody for 25 min on ice in the dark. Unstained controls were resuspended in cold FACS buffer and incubated likewise. Afterwards, cells were washed twice with cold FACS buffer, resuspended in FACS buffer, and analyzed with FACS Aria III (BD Bioscience). Gates were set to remove debris and select single cells (Fig. S2, Additional File 2). Median fluorescence intensities were analyzed to obtain relative surface receptor levels. Post-measurement analysis was performed with FlowJo^™^ version 10.8.1 (BD Life Sciences).

### Immunocytochemistry

To determine the presence of SP-C and the survival of differentiated THP-1 cells at the ALI, immunocytochemical staining and fluorescence microscopy were used. A549 cells in co-culture with wild type, *CASP1*^−/−^, or *NLRP3*^−/−^ THP-1 cells or without THP-1 cells were cultivated at the ALI for 48 h. Subsequently the cultures were fixed with 4% paraformaldehyde in PBS. After blocking with 10% normal goat serum (NGS) and 3% BSA in PBS, the co-cultures were incubated with SP-C (1:40; Thermo Fisher Scientific, #PA5-71680) and CD45 (1:500; BD Biosciences, #561863) primary antibodies in blocking solution overnight at 4 °C. After rinsing 5x with 3% BSA in PBS, the co-cultures were incubated with Alexa488 goat-anti-rabbit and Alexa596 goat-anti-mouse (both 6.7 µg/mL; Thermo Fisher Scientific) secondary antibodies and Hoechst 33342 (0.5 µg/mL) in blocking solution for 1 h at room temperature. After rinsing 5x with 3% BSA in PBS, 1x with PBS, and 1x with dH_2_O, the co-cultures were placed into a drop of Prolong Gold Antifade Mountant (Thermo Fisher Scientific) on a microscopy glass slide. Fluorescence images were acquired at 100 x magnification using a Zeiss Axio Imager.M2 fluorescence microscope. Simultaneously, images of autofluorescent plastic slides (Chroma) were acquired for flat field correction. Flat field corrections for each channel, for CD45 rolling ball background subtractions with a radius of 40, and merging of images were done with ImageJ (version 1.53t) macros (Supplementary Material 1, Additional File 9). Simultaneously, control stainings without first antibodies or without secondary antibodies were performed (Fig. S7, Additional File 10).

### Air-liquid interface exposure procedure

Dusts were suspended and sonicated as described above. As a negative control, endotoxin-free H_2_O containing 1.25% PBS was sonicated under the same conditions. The suspensions were warmed to 37 °C and vortexed vigorously before use. For exposure at the ALI, the Vitrocell Cloud 12α system was used. Dust suspensions were nebulized using a Vitrocell 9–12 μm nebulizer to which a 600 Hz square signal was applied using a Velleman PCSU200 Oscilloscope. Through preliminary experiments using suspensions spiked with fluorescein, the uniform deposition in all used wells could be confirmed. Controls and LPS were nebulized using 4–6 μm nebulizers.

The Cloud system was loaded as exemplified in Fig. S8A, Additional File 11. The bigger exposure chamber consisted of nine wells and the smaller control chamber of three wells. The top-left well of the exposure chamber was equipped with a Vitrocell sQCM for online dosimetry. To the middle well and one other well of the exposure chamber, 3 mL PBS was added. The middle well was left empty. To analyze the deposited dusts via SEM, a TEM grid was placed into a stainless-steel insert which was placed onto PBS. To the residual six wells of the exposure chamber and the three wells of the control chamber, 3 mL A549 medium containing 25 mM HEPES was added and inserts with co-cultures were placed on top. For each dose or genotype, one corner well and one side well were used.

After loading, exposure was performed as illustrated in Fig. S8B, Additional File 11. The aerosol chamber was mounted and humidity in the aerosol chamber was allowed to saturate for 30 min. Subsequently, 500 µL of an SD or DQ12 suspension corresponding to a deposited concentration of about 10 µg/cm² was nebulized in the exposure chamber. Simultaneously, 236 µL control solution was nebulized in the control chamber. The sedimentation process was observed based on the sQCM signal. When sedimentation was completed after about 30 min, the aerosol chamber was dismounted and the sQCM signal was allowed to stabilize. Optionally, to test different doses, transwell inserts were transferred back from the Cloud system to 12-well plates with 1.2 mL fresh A549 medium. Per two exposed co-cultures, one control co-culture was removed from the Cloud system. Then the procedure was repeated twice more to reach doses of about 20 µg/cm² and about 30 µg/cm². To expose co-cultures to LPS as a positive control directly afterwards, the aerosol chamber was cleaned and the required wells of the exposure chamber were rinsed and filled with fresh A549 medium containing 25 mM HEPES. The aerosol chamber was mounted and humidity in the aerosol chamber was allowed to saturate for 30 min. Subsequently, 200 µL of 262 µg/mL LPS in endotoxin-free H_2_O containing 1.25% PBS corresponding to a deposited LPS concentration of about 250 ng/cm² was nebulized in the exposure chamber. Sedimentation was observed based on the sQCM signal, the aerosol chamber was removed, the sQCM signal was allowed to stabilize, and co-cultures were transferred back to 12-well plates with 1.2 mL fresh A549 medium.

A volume of 600 µL of 12% BSA in HBSS containing CaCl_2_ and MgCl_2_ was added to the apical side of each co-culture 30 min before the end of exposure time. BSA served to inhibit interferences with ELISAs caused by the absorbance of cytokines on the particle surfaces. Exposure was terminated 24 h after the first nebulization of SD or DQ12. Of the apical and basolateral supernatants, 2 × 20 µL were collected for the ToxiLight assay. The residual supernatants were stored at -20 °C for ELISAs. The inserts with cells were rinsed with PBS twice, lysed in TRIzol reagent, and stored at -80 °C for RNA isolation.

To assess the interference of SD with cytokine ELISAs, instead of transwell inserts with cells, stainless steel inserts filled with a mixture of recombinant cytokines were used. Each stainless steel insert was filled with 200 µL of A549 medium substituted with 750 pg/mL IL-1β, 600 pg/mL IL-6, 1920 pg/mL IL-8, and 960 pg/mL TNFα. In the exposure chamber and control chamber, an SD suspension and control solution were nebulized onto the cytokine mixtures, respectively, following the procedure described above. After incubation for 23.5 h, 1.0 mL of 14% BSA in HBSS containing CaCl_2_ and MgCl_2_ was added to each insert. After a further 30 min, the mixtures were collected and the cytokine concentrations were analyzed via ELISA.

### ToxiLight cytotoxicity assay

To assess cytotoxicity, the Lonza ToxiLight assay measuring adenylate kinase release was performed according to the supplier’s instructions. Briefly, 2 × 20 µL of apical and basolateral supernatants were transferred to white 96-well plates. To each well, 100 µL adenylate kinase detection reagent mix was added. Following incubation for 15–30 min, luminescence was measured using a TECAN Spark or TECAN Infinite 200 Pro reader. As positive control, 15 min before the end of exposure, 120 µL and 60 µL of 5% Triton X-100 were added to the basolateral and apical side of each transwell, respectively.

### Gene expression analysis

The expressions of *HMOX1*, *APE1/REF1*, *GGCS*, *NQO1*, *IL1B*, *IL6*, *IL8*, *TNFA,* and *IL18* were assessed by qRT-PCR. RNA was isolated from TRIzol lysates as described previously [21]. RNA quantification, DNase I digestion, reverse transcription, and qRT-PCR were performed as described previously [101]. Briefly, the optical density of RNA was measured at 260 and 280 nm to determine the concentration. A total of 1.5 µg RNA per sample was treated with amplification grade DNase I. Of this RNA, 2 × 0.5 µg was reverse transcribed using the iScript™ cDNA synthesis kit. A no reverse transcriptase control (nRTc) with 0.5 µg RNA was performed in parallel to control for residual DNA. The cDNA and nRTc were diluted in nuclease-free H_2_O by factor 15. When less RNA was available, the amount of RNA and the dilution factor were decreased proportionally. The primers listed in Table S3, Additional File 12, were used. Actin beta (*ACTB*) and glyceraldehyde 3phosphate dehydrogenase (*GAPDH*) were analyzed as reference genes. C_T_ values were determined using the Bio-Rad iQ5 software (v2.1) or QuantStudio™ Design & Analysis Software and corrected for primer efficiencies. Exposure-dependent changes in the gene expression were calculated using the ΔΔC_T_ method [102]. Relatively large standard deviations can be explained by variability of the constitutive gene expression between the independent experiments. This did not affect the statistical analysis since the experimental run was included as a random factor in the mixed-effects models (see Statistics).

### Cytokine quantification by ELISA

The concentrations of IL-1β, IL-6, IL-8, TNFα, and IL-18 in apical and basolateral supernatants were analyzed using R&D systems DuoSet ELISA kits as described previously [103]. Briefly, high-protein-binding 96-well plates were coated with primary antibody, blocked with BSA, and incubated with 100 µL sample. Consecutively, detection antibody, horseradish peroxidase, and BioRad TMB Peroxidase EIA Substrate were incubated. The color reaction was stopped with H_2_SO_4_ and absorbance was measured at 450 and 540 nm. The standard curve was plotted using a four-parameter log fit. In case the measured concentrations were below the limit of detection (LOD), half the LODs, i.e. 1.95 pg/mL, 4.69 pg/mL, 15.63 pg/mL, 7.81 pg/mL, and 5.86 pg/mL for IL-1β, IL-6, IL-8, TNFα, and IL-18, respectively, were used for calculations and statistics. Relatively large standard deviations can be explained by variability of the constitutive cytokine releases between the independent experiments. This did not affect the statistical analysis since the experimental run was included as a random factor in the mixed-effects models (see Statistics).

### Statistics

Histograms of fluorescence intensities from flow cytometry were created with FlowJo™ version 10.8.1. For all other data, GraphPad Prism version 9.1.0 was used for the visualization of means and standard deviation. Microsoft Excel was used to calculate ΔC_T_ values, ΔΔC_T_ values, fold changes, mean values, and standard deviations. Firstly, for each independent experiment, mean values were calculated from the biological replicates. These mean values from at least three independent experiments were then used for further calculations. For the gene expression analyses, ΔC_T_ values were used for statistics. For visualization, mean values from ΔΔC_T_ values with standard deviations from ΔC_T_ values were converted into fold changes. To test statistical significance of the flow cytometry data, a two-way ANOVA with Tukey’s post hoc test was calculated in GraphPad Prism. For all other data, mixed-effects models with Šídák’s post hoc test were applied in R version 4.1.2 to test statistical significance. In the mixed-effects models, exposure and, where applicable genotype, were applied as fixed factors. The experimental run was applied as random factor. *p*-values of ≤ 0.05 were considered statistically significant.

### Electronic supplementary material

Below is the link to the electronic supplementary material.

**Additional file 1: “Fig. S1.jpg”**. Representative images of nebulized DQ12 and Saharan dust used for size determination. Suspensions of DQ12 quartz dust (**A**, **B**) and Saharan dust (**C**, **D**) in endotoxin-free H_2_O containing 1.25% PBS were sonicated and nebulized onto 0.1 μm pore-size nucleopore filters. Images were obtained at a nominal magnification of 5,000 x (pixel size: 6.2 nm). Images **A** and **C** show excerpts from images used for size determination. Images **B** and **D** show the same areas after size determination using ImageJ.

**Additional file 2: “Fig. S2.jpg”**. Gating strategy of flow cytometry analysis. The first gate (SSC-A vs. FSC-A) was set to remove debris. Then doublets were discriminated (linear FSC-H vs. FSC-A) and median fluorescence intensity was determined in the APC-A channel. A representative dot plot is shown for differentiated unstained THP-1 wild type cells. The gating strategy is identical for all samples. SSC: side scatter, FSC: forward scatter, APC: allophycocyanin, A: area, H: height.

**Additional file 3: “Fig. S3.tif”**. IL-1β release from THP-1 cells submerged. In parallel to ALI co-culture experiments (Figs. 3 and 7), the same wild type, *CASP1*^−/−^, and *NLRP3*^−/−^ THP-1 cells were seeded in 24-well plates and exposed to 10 ng/mL LPS submerged for 24 h. In each experiment, one biological replicated was tested per group. The IL-1β concentrations in the supernatants were measured via ELISA. These submerged experiments were performed to confirm the genotypes of the THP-1 cells via comparison of the results to data from a previous study [55]. Depicted are means and standard deviations of *N* = 4 independent experiments. n.d.: not detected.

**Additional file 4: “Fig. S4.jpg”**. Exemplary scanning electron microscopy images of Saharan dust and DQ12 quartz dust deposited on transmission electron microscopy grids during air-liquid interface exposure of co-cultures. A stainless steel insert with a TEM grid was placed into the Vitrocell Cloud 12α in parallel to ALI co-cultures and loaded with Saharan dust or DQ12, which were nebulized and deposited onto the inserts. Deposited doses of 10.9 µg/cm² SD (**A**-**C**), 10.4 µg/cm² DQ12 (**D**-**F**), 30.8 µg/cm² SD (**G**-**J**), and 30.1 µg/cm² DQ12 (**K**-**M**) were measured via quartz crystal microbalance. Images were obtained at nominal magnifications of 61 x (pixel size: 1.02 μm) (**A**, **D**, **G**, **K**), 2.5 kx (pixel size: 24.8 nm) (**B**, **E**, **H**, **L**), and 10 kx (pixel size: 6.2 nm) (**C**, **F**, **J**, **M**).

**Additional file 5: “Table S1.docx”**. Depositions of DQ12, LPS, and SD in µg/cm² measured with Vitrocell sQCM. (belonging to Figs. 4, 5, and 6). Depositions from single experiments are presented with their means and standard deviations (St. dev.). For DQ12 and SD, accumulative doses after first (1x), second (2x), and third (3x) nebulization are shown.

**Additional file 6: “Fig. S5.tif”**. Interference with cytokine ELISAs. In a previous study, we found that SD but not DQ12 interfered with enzyme-linked immuno-sorbent assays (ELISAs) [21]. To assess the interaction of nebulized SD with IL-1β, IL-6, IL-8, and TNFα ELISAs, we nebulized and deposited SD on mixtures of recombinant cytokines (*N* = 1). The control was tested in triplicate, and each dust concentration in duplicates. Cytokine concentrations were determined after incubation for 24 h. SD weakly decreased the recovery of IL-6 by about 15% at the highest tested concentration of 27.8 µg/cm². SD concentrations of 9.2–27.8 µg/cm² decreased the recovery of IL-8 more strongly and dose-dependently by about 45–75%. SD barely affected the recovery of TNFα. For IL-1β, the interference could not be analyzed. Even in the negative control, IL-1β could not be detected after incubation in stainless steel inserts for 24 h. Considering the much lower interference of SD with IL-1β than with IL-8 in submerged experiments [21], at most a weak interference of SD with IL-1β is expected. In addition, in the interference experiment, SD was nebulized into a simulated apical compartment whereas basolateral supernatants were analyzed for cytokine release. Thus, even lower interferences in the ALI co-culture exposures than in this simulation are expected.

**Additional file 7: “Table S2.docx”**. Depositions of SD and LPS in µg/cm² measured with Vitrocell sQCM (belonging to Fig. 7). Depositions from single experiments are presented with their means and standard deviations (St. dev.). For SD, the accumulative doses after the third (3x) nebulization are shown.

**Additional file 8: “Fig. S6.tif”**. Air-liquid interface co-culture preparation. On day 1, A549 cells are seeded on the apical side of transwell inserts. On day 2, after incubation for 24 h, apical and basolateral medium are changed. Simultaneously, THP-1 cells are differentiated to macrophage-like cells through incubation with phorbol 12-myristate-13-acetate (PMA) for 24 h. On day 3, the differentiated THP-1 cells are detached with accutase and seeded on the apical side of the confluent A549 layer. Following attachment for 2 h, the apical medium is removed to initiate air-liquid interface (ALI) culture. On day 4, following 22–26 h of culture at the ALI, co-cultures are exposed at the ALI using a Vitrocell Cloud 12α. Co-cultures are exposed for 24 h.

**Additional file 9: “Supplementary material 1.docx”**. ImageJ macro codes. Codes used to perform flat field corrections (**A**-**D**), rolling ball background subtraction (**C**, **D**), and merging (**D**) for images depicted in Fig. 3 and Fig. S7.

**Additional file 10: “Fig. S7.tif”**. Control staining for surfactant protein C (SP-C) and CD45. Co-cultures of A549 cells with wild type THP-1 cells were fixed after 48 h of cultivation at the ALI. Nuclei were stained with Hoechst 33342. Immunostaining of SP-C and CD45 was performed using either primary and secondary antibodies, primary antibodies only, or secondary antibodies only. Representative images were obtained at 100 x magnification.

**Additional file 11: “Fig. S8.tif”**. Exemplary loading pattern and procedure of Vitrocell Cloud 12α experiments. **A**: The bigger exposure chamber consists of nine wells and the smaller control chamber of three wells. The Vitrocell quartz crystal microbalance (sQCM) was installed in the top-left well. The middle well and the left-middle well of the exposure chamber were filled with 3 mL PBS. Optionally, a stainless-steel insert with a transmission electron microscopy (TEM) grid was placed into the middle-left position. The other wells were filled with 3 mL A549 medium containing 25 mM HEPES and co-culture inserts were placed on top. For each dose, one corner and one middle well were used. **B**: After each nebulization of SD or DQ12, sedimentation took about 30 min. The aerosol chamber was dismounted and optionally co-cultures could be removed from the Cloud system to obtain different doses. Subsequently, the aerosol chamber was mounted again and humidity was allowed to saturate for 30 min. This procedure was repeated twice. Directly after the third sedimentation of DQ12 and removing exposed co-cultures, the aerosol chamber and required wells for exposure to lipopolysaccharide (LPS) were cleaned and loaded. LPS was nebulized. All co-cultures were incubated until 24 h after the first nebulization of particles had passed.

**Additional file 12: “Table S3.docx”**. qPCR primer pairs. Sequences and concentrations of the used qPCR primers and amplicon lengths and efficiencies of the used qPCR Primer pairs on both used devices.

## Data Availability

The datasets generated and/or analyzed during the current study are available in the Mendeley Data repository, doi: 10.17632/znd6v266cx.2.

## References

[1] Kotsyfakis M, Zarogiannis SG, Patelarou E (2019). The health impact of Saharan dust exposure. Int J Occup Med Environ Health.

[2] Stafoggia M, Zauli-Sajani S, Pey J, Samoli E, Alessandrini E, Basagana X (2016). Desert Dust outbreaks in Southern Europe: contribution to Daily PM(1)(0) concentrations and short-term associations with Mortality and Hospital admissions. Environ Health Perspect.

[3] Trianti SM, Samoli E, Rodopoulou S, Katsouyanni K, Papiris SA, Karakatsani A (2017). Desert dust outbreaks and respiratory morbidity in Athens, Greece. Environ Health.

[4] Johnston F, Hanigan I, Henderson S, Morgan G, Bowman D (2011). Extreme air pollution events from bushfires and dust Storms and their association with mortality in Sydney, Australia 1994–2007. Environ Res.

[5] Kashima S, Yorifuji T, Bae S, Honda Y, Lim Y-H, Hong Y-C (2016). Asian dust effect on cause-specific mortality in five cities across South Korea and Japan. Atmos Environ.

[6] He M, Ichinose T, Yoshida S, Nishikawa M, Mori I, Yanagisawa R (2010). Airborne Asian sand dust enhances murine lung eosinophilia. Inhal Toxicol.

[7] Ichinose T, Nishikawa M, Takano H, Sera N, Sadakane K, Mori I (2005). Pulmonary toxicity induced by intratracheal instillation of Asian yellow dust (Kosa) in mice. Environ Toxicol Pharmacol.

[8] Wilfong ER, Lyles M, Rietcheck RL, Arfsten DP, Boeckman HJ, Johnson EW (2011). The acute and long-term effects of Middle East sand particles on the rat airway following a single intratracheal instillation. J Toxicol Env Heal a.

[9] Taylor K, Foster ML, Law JM, Centeno JA, Fornero E, Henderson MS (2013). Assessment of geographical variation in the respiratory toxicity of desert dust particles. Inhal Toxicol.

[10] Ghio AJ, Kummarapurugu ST, Tong H, Soukup JM, Dailey LA, Boykin E (2014). Biological effects of desert dust in respiratory epithelial cells and a murine model. Inhal Toxicol.

[11] Keil DE, Buck B, Goossens D, McLaurin B, Murphy L, Leetham-Spencer M (2018). Nevada desert dust with heavy metals suppresses IgM antibody production. Toxicol Rep.

[12] Shin SH, Ye MK, Hwang YJ, Kim ST (2013). The effect of Asian sand dust-activated respiratory epithelial cells on activation and migration of eosinophils. Inhal Toxicol.

[13] He M, Ichinose T, Song Y, Yoshida Y, Arashidani K, Yoshida S (2013). Effects of two Asian sand dusts transported from the dust source regions of Inner Mongolia and northeast China on murine lung eosinophilia. Toxicol Appl Pharmacol.

[14] Rodriguez-Navarro C, di Lorenzo F, Elert K (2018). Mineralogy and physicochemical features of Saharan dust wet deposited in the Iberian Peninsula during an extreme red rain event. Atmos Chem Phys.

[15] Schlesinger P, Mamane Y, Grishkan I (2006). Transport of microorganisms to Israel during Saharan dust events. Aerobiologia.

[16] Ginoux P, Prospero JM, Gill TE, Hsu NC, Zhao M. Global-scale attribution of anthropogenic and natural dust sources and their emission rates based on MODIS Deep Blue aerosol products. Rev Geophys. 2012;50(3). 10.1029/2012rg000388.

[17] Ortiz-Martinez MG, Rodriguez-Cotto RI, Ortiz-Rivera MA, Pluguez-Turull CW, Jimenez-Velez BD (2015). Linking endotoxins, African dust PM10 and Asthma in an Urban and Rural Environment of Puerto Rico. Mediat Inflamm.

[18] Rodriguez-Cotto RI, Ortiz-Martinez MG, Jimenez-Velez BD (2015). Organic extracts from African dust Storms stimulate oxidative stress and induce inflammatory responses in human lung cells through Nrf2 but not NF-kappaB. Environ Toxicol Pharmacol.

[19] Rodriguez-Cotto RI, Ortiz-Martinez MG, Rivera-Ramirez E, Mendez LB, Davila JC, Jimenez-Velez BD (2013). African dust Storms reaching Puerto Rican Coast stimulate the secretion of IL-6 and IL-8 and cause cytotoxicity to human bronchial epithelial cells (BEAS-2B). Health (Irvine Calif).

[20] Val S, Liousse C, Doumbia el HT, Galy-Lacaux C, Cachier H, Marchand N (2013). Physico-chemical characterization of African urban aerosols (Bamako in Mali and Dakar in Senegal) and their toxic effects in human bronchial epithelial cells: description of a worrying situation. Part Fibre Toxicol.

[21] Bredeck G, Busch M, Rossi A, Stahlmecke B, Fomba KW, Herrmann H (2023). Inhalable saharan dust induces oxidative stress, NLRP3 inflammasome activation, and inflammatory cytokine release. Environ Int.

[22] Bauernfeind FG, Horvath G, Stutz A, Alnemri ES, MacDonald K, Speert D (2009). Cutting edge: NF-kappaB activating pattern recognition and cytokine receptors license NLRP3 inflammasome activation by regulating NLRP3 expression. J Immunol.

[23] Kahlenberg JM, Lundberg KC, Kertesy SB, Qu Y, Dubyak GR (2005). Potentiation of caspase-1 activation by the P2 × 7 receptor is dependent on TLR signals and requires NF-kappaB-driven protein synthesis. J Immunol.

[24] Jo EK, Kim JK, Shin DM, Sasakawa C (2016). Molecular mechanisms regulating NLRP3 inflammasome activation. Cell Mol Immunol.

[25] Thornberry NA, Bull HG, Calaycay JR, Chapman KT, Howard AD, Kostura MJ (1992). A novel heterodimeric cysteine protease is required for interleukin-1 beta processing in monocytes. Nature.

[26] Martinon F, Burns K, Tschopp J (2002). The inflammasome: a molecular platform triggering activation of inflammatory caspases and processing of proIL-beta. Mol Cell.

[27] Jobin C, Haskill S, Mayer L, Panja A, Sartor RB (1997). Evidence for altered regulation of I kappa B alpha degradation in human colonic epithelial cells. J Immunol.

[28] Parikh AA, Salzman AL, Kane CD, Fischer JE, Hasselgren PO (1997). IL-6 production in human intestinal epithelial cells following stimulation with IL-1 beta is associated with activation of the transcription factor NF-kappa B. J Surg Res.

[29] Bonizzi G, Piette J, Merville MP, Bours V (1997). Distinct signal transduction pathways mediate nuclear factor-kappab induction by IL-1beta in epithelial and lymphoid cells. J Immunol.

[30] Grailer JJ, Canning BA, Kalbitz M, Haggadone MD, Dhond RM, Andjelkovic AV (2014). Critical role for the NLRP3 inflammasome during acute lung injury. J Immunol.

[31] Yang HH, Duan JX, Liu SK, Xiong JB, Guan XX, Zhong WJ (2020). A COX-2/sEH dual inhibitor PTUPB alleviates lipopolysaccharide-induced acute lung injury in mice by inhibiting NLRP3 inflammasome activation. Theranostics.

[32] Zhang Y, Li X, Grailer JJ, Wang N, Wang M, Yao J (2016). Melatonin alleviates acute lung injury through inhibiting the NLRP3 inflammasome. J Pineal Res.

[33] Cassel SL, Eisenbarth SC, Iyer SS, Sadler JJ, Colegio OR, Tephly LA (2008). The Nalp3 inflammasome is essential for the development of silicosis. Proc Natl Acad Sci U S A.

[34] Dostert C, Petrilli V, Van Bruggen R, Steele C, Mossman BT, Tschopp J (2008). Innate immune activation through Nalp3 inflammasome sensing of asbestos and silica. Science.

[35] Ghayur T, Banerjee S, Hugunin M, Butler D, Herzog L, Carter A (1997). Caspase-1 processes IFN-gamma-inducing factor and regulates LPS-induced IFN-gamma production. Nature.

[36] Davis GS, Pfeiffer LM, Hemenway DR, Rincon M (2006). Interleukin-12 is not essential for silicosis in mice. Part Fibre Toxicol.

[37] Mohebbi I, Rad IA, Bagheri M (2013). Interleukin-18, interleukin-8, and CXCR2 and the risk of silicosis. Toxicol Ind Health.

[38] Jessop F, Hamilton RF, Rhoderick JF, Shaw PK, Holian A (2016). Autophagy deficiency in macrophages enhances NLRP3 inflammasome activity and chronic lung Disease following silica exposure. Toxicol Appl Pharmacol.

[39] Stephens KE, Ishizaka A, Larrick JW, Raffin TA (1988). Tumor necrosis factor causes increased pulmonary permeability and edema. Comparison to septic acute lung injury. Am Rev Respir Dis.

[40] Piguet PF, Collart MA, Grau GE, Sappino AP, Vassalli P (1990). Requirement of tumour necrosis factor for development of silica-induced pulmonary fibrosis. Nature.

[41] Ohlinger K, Kolesnik T, Meindl C, Galle B, Absenger-Novak M, Kolb-Lenz D (2019). Air-liquid interface culture changes surface properties of A549 cells. Toxicol in Vitro.

[42] Blank F, Rothen-Rutishauser BM, Schurch S, Gehr P (2006). An optimized in vitro model of the respiratory tract wall to study particle cell interactions. J Aerosol Med.

[43] Wu J, Wang Y, Liu G, Jia Y, Yang J, Shi J (2017). Characterization of air-liquid interface culture of A549 alveolar epithelial cells. Braz J Med Biol Res.

[44] Herseth JI, Volden V, Schwarze PE, Lag M, Refsnes M (2008). IL-1beta differently involved in IL-8 and FGF-2 release in crystalline silica-treated lung cell co-cultures. Part Fibre Toxicol.

[45] Standiford TJ, Kunkel SL, Basha MA, Chensue SW, Lynch JP, Toews GB (1990). Interleukin-8 gene expression by a pulmonary epithelial cell line. A model for cytokine networks in the lung. J Clin Invest.

[46] Li S, Sun Z, Chen T, Pan J, Shen Y, Chen X (2019). The role of mir-431-5p in regulating pulmonary surfactant expression in vitro. Cell Mol Biol Lett.

[47] Hussell T, Bell TJ (2014). Alveolar macrophages: plasticity in a tissue-specific context. Nat Rev Immunol.

[48] Murakami S, Iwaki D, Mitsuzawa H, Sano H, Takahashi H, Voelker DR (2002). Surfactant protein A inhibits peptidoglycan-induced Tumor necrosis factor-alpha secretion in U937 cells and alveolar macrophages by direct interaction with toll-like receptor 2. J Biol Chem.

[49] Yamada C, Sano H, Shimizu T, Mitsuzawa H, Nishitani C, Himi T (2006). Surfactant protein A directly interacts with TLR4 and MD-2 and regulates inflammatory cellular response. Importance of supratrimeric oligomerization. J Biol Chem.

[50] Augusto LA, Synguelakis M, Espinassous Q, Lepoivre M, Johansson J, Chaby R (2003). Cellular antiendotoxin activities of lung surfactant protein C in lipid vesicles. Am J Resp Crit Care.

[51] Gehr P, Green FH, Geiser M, Im Hof V, Lee MM, Schurch S (1996). Airway surfactant, a primary defense barrier: mechanical and immunological aspects. J Aerosol Med.

[52] Wallace WE, Keane MJ, Mike PS, Hill CA, Vallyathan V, Regad ED (1992). Contrasting respirable quartz and kaolin retention of lecithin surfactant and expression of membranolytic activity following phospholipase A2 digestion. J Toxicol Environ Health.

[53] Emerson RJ, Davis GS (1983). Effect of alveolar lining material-coated silica on rat alveolar macrophages. Environ Health Perspect.

[54] Pavan C, Rabolli V, Tomatis M, Fubini B, Lison D (2014). Why does the hemolytic activity of silica predict its pro-inflammatory activity?. Part Fibre Toxicol.

[55] Busch M, Ramachandran H, Wahle T, Rossi A, Schins RPF (2022). Investigating the role of the NLRP3 inflammasome pathway in Acute Intestinal inflammation: use of THP-1 knockout cell lines in an Advanced Triple Culture Model. Front Immunol.

[56] Thomassen MJ, Antal JM, Connors MJ, Meeker DP, Wiedemann HP (1994). Characterization of exosurf (surfactant)-mediated suppression of stimulated human alveolar macrophage cytokine responses. Am J Respir Cell Mol Biol.

[57] Kanj RS, Kang JL, Castranova V (2006). Interaction between primary alveolar macrophages and primary alveolar type II cells under basal conditions and after lipopolysaccharide or quartz exposure. J Toxicol Environ Health A.

[58] Kuan SF, Rust K, Crouch E (1992). Interactions of surfactant protein D with bacterial lipopolysaccharides. Surfactant protein D is an Escherichia coli-binding protein in bronchoalveolar lavage. J Clin Invest.

[59] McNeely TB, Coonrod JD (1993). Comparison of the opsonic activity of human surfactant protein A for Staphylococcus aureus and Streptococcus pneumoniae with rabbit and human macrophages. J Infect Dis.

[60] Chow JC, Young DW, Golenbock DT, Christ WJ, Gusovsky F (1999). Toll-like receptor-4 mediates lipopolysaccharide-induced signal transduction. J Biol Chem.

[61] International Commission on Radiological Protection (ICRP) (1994). Human respiratory tract model for Radiological Protection. ICRP publication 66. Ann ICRP.

[62] Friesen A, Fritsch-Decker S, Hufnagel M, Mulhopt S, Stapf D, Hartwig A (2022). Comparing alpha-quartz-Induced cytotoxicity and Interleukin-8 release in Pulmonary Mono- and co-cultures exposed under submerged and air-liquid interface conditions. Int J Mol Sci.

[63] van Berlo D, Knaapen AM, van Schooten FJ, Schins RP, Albrecht C (2010). NF-kappaB dependent and Independent mechanisms of quartz-induced proinflammatory activation of lung epithelial cells. Part Fibre Toxicol.

[64] Schins RP, McAlinden A, MacNee W, Jimenez LA, Ross JA, Guy K (2000). Persistent depletion of I kappa B alpha and interleukin-8 expression in human pulmonary epithelial cells exposed to quartz particles. Toxicol Appl Pharmacol.

[65] United States Department of Labor Occupational Safety and Health Administration. 1910.1053 - Respirable crystalline silica.https://www.osha.gov/laws-regs/regulations/standardnumber/1910/1910.1053. Accessed 10 Apr 2023.

[66] European Parliament. Directive (EU) 2017/2398 of the European Parliament and of the Council of 12 December 2017 amending Directive 2004/37/EC on the protection of workers from the risks related to exposure to carcinogens or mutagens at work (Text with EEA relevance).2017. https://eur-lex.europa.eu/eli/dir/2017/2398/oj. Accessed 12 Apr 2023.

[67] Gama C, Tchepel O, Baldasano JM, Basart S, Ferreira J, Pio C (2015). Seasonal patterns of Saharan dust over Cape Verde – a combined approach using observations and modelling. Tellus B.

[68] Garrison VH, Majewski MS, Konde L, Wolf RE, Otto RD, Tsuneoka Y. Inhalable desert dust, urban emissions, and potentially biotoxic metals in urban saharan–sahelian air. Sci Total Environ. 2014;500–1. 10.1016/j.scitotenv.2014.08.106. 10.1016/j.scitotenv.2014.08.106 25243921

[69] Bitterle E, Karg E, Schroeppel A, Kreyling WG, Tippe A, Ferron GA (2006). Dose-controlled exposure of A549 epithelial cells at the air-liquid interface to airborne ultrafine carbonaceous particles. Chemosphere.

[70] Loret T, Peyret E, Dubreuil M, Aguerre-Chariol O, Bressot C, le Bihan O (2016). Air-liquid interface exposure to aerosols of poorly soluble nanomaterials induces different biological activation levels compared to exposure to suspensions. Part Fibre Toxicol.

[71] Skuland T, Lag M, Gutleb AC, Brinchmann BC, Serchi T, Ovrevik J, et al. Pro-inflammatory effects of crystalline- and nano-sized non-crystalline silica particles in a 3D alveolar model. Part Fibre Toxicol. 2020;17(1:13). 10.1186/s12989-020-00345-3. 10.1186/s12989-020-00345-3 PMC7175518 32316988

[72] Alfaro-Moreno E, Nawrot TS, Vanaudenaerde BM, Hoylaerts MF, Vanoirbeek JA, Nemery B (2008). Co-cultures of multiple cell types mimic pulmonary cell communication in response to urban PM10. Eur Respir J.

[73] Wang G, Zhang X, Liu X, Zheng J (2020). Co-culture of human alveolar epithelial (A549) and macrophage (THP-1) cells to study the potential toxicity of ambient PM2.5: a comparison of growth under ALI and submerged conditions. Toxicol Res (Camb).

[74] Russell WMS, Burch RL. The principles of Humane experimental technique. Methuen; 1959.

[75] Naota M, Mukaiyama T, Shimada A, Yoshida A, Okajima M, Morita T (2010). Pathological study of acute pulmonary toxicity induced by intratracheally instilled Asian sand dust (kosa). Toxicol Pathol.

[76] Ren Y, Ichinose T, He M, Song Y, Yoshida Y, Yoshida S (2014). Enhancement of OVA-induced murine lung eosinophilia by co-exposure to contamination levels of LPS in Asian sand dust and heated dust. Allergy Asthma Cl Im.

[77] Kim K, Kim S-D, Shin T-H, Bae C-S, Ahn T, Shin S-S (2021). Respiratory and systemic toxicity of Inhaled Artificial Asian Sand Dust in pigs. Life.

[78] Kim YH, Kim KS, Kwak NJ, Lee KH, Kweon SA, Lim Y (2003). Cytotoxicity of yellow sand in lung epithelial cells. J Biosci.

[79] Stern RA, Mahmoudi N, Buckee CO, Schartup AT, Koutrakis P, Ferguson ST (2021). The Microbiome of size-fractionated Airborne particles from the Sahara Region. Environ Sci Technol.

[80] Endes C, Schmid O, Kinnear C, Mueller S, Camarero-Espinosa S, Vanhecke D (2014). An in vitro testing strategy towards mimicking the inhalation of high aspect ratio nanoparticles. Part Fibre Toxicol.

[81] Ovrevik J, Refsnes M, Schwarze P, Lag M (2008). The ability of oxidative stress to mimic quartz-induced chemokine responses is lung cell line-dependent. Toxicol Lett.

[82] Panas A, Comouth A, Saathoff H, Leisner T, Al-Rawi M, Simon M (2014). Silica nanoparticles are less toxic to human lung cells when deposited at the air-liquid interface compared to conventional submerged exposure. Beilstein J Nanotech.

[83] Geng H, Meng Z, Zhang Q (2005). Effects of blowing sand fine particles on plasma membrane permeability and fluidity, and intracellular calcium levels of rat alveolar macrophages. Toxicol Lett.

[84] Ghiazza M, Scherbart AM, Fenoglio I, Grendene F, Turci F, Martra G (2011). Surface iron inhibits quartz-induced cytotoxic and inflammatory responses in alveolar macrophages. Chem Res Toxicol.

[85] Albrecht C, Schins RP, Hohr D, Becker A, Shi T, Knaapen AM (2004). Inflammatory time course after quartz instillation: role of Tumor necrosis factor-alpha and particle surface. Am J Respir Cell Mol Biol.

[86] Nakano-Narusawa Y, Yokohira M, Yamakawa K, Saoo K, Imaida K, Matsuda Y (2020). Single Intratracheal Quartz Instillation Induced chronic inflammation and tumourigenesis in rat lungs. Sci Rep.

[87] Goodarzi M, Azizi S, Koupaei MJ, Moshkelani S (2014). Pathologic findings of Anthraco-silicosis in the lungs of one Humped camels (Camelus dromedarius) and its role in the occurrence of Pneumonia. Kafkas Univ Vet Fak.

[88] Hansen HJ, Jama FM, Nilsson C, Norrgren L, Abdurahman OS (1989). Silicate pneumoconiosis in camels (Camelus dromedarius L). J Vet Med A.

[89] Midtbo K, Eklund D, Sarndahl E, Persson A (2020). Molecularly distinct NLRP3 inducers mediate diverse ratios of Interleukin-1beta and Interleukin-18 from human monocytes. Mediators Inflamm.

[90] Erener S, Petrilli V, Kassner I, Minotti R, Castillo R, Santoro R (2012). Inflammasome-activated caspase 7 cleaves PARP1 to enhance the expression of a subset of NF-kappaB target genes. Mol Cell.

[91] Falvo JV, Tsytsykova AV, Goldfeld AE (2010). Transcriptional control of the TNF gene. Curr Dir Autoimmun.

[92] Martinon F, Burns K, Tschopp J (2002). The Inflammasome. Mol Cell.

[93] Poyet JL, Srinivasula SM, Tnani M, Razmara M, Fernandes-Alnemri T, Alnemri ES (2001). Identification of Ipaf, a human caspase-1-activating protein related to Apaf-1. J Biol Chem.

[94] Burckstummer T, Baumann C, Bluml S, Dixit E, Durnberger G, Jahn H (2009). An orthogonal proteomic-genomic screen identifies AIM2 as a cytoplasmic DNA sensor for the inflammasome. Nat Immunol.

[95] Grenier JM, Wang L, Manji GA, Huang WJ, Al-Garawi A, Kelly R (2002). Functional screening of five PYPAF family members identifies PYPAF5 as a novel regulator of NF-kappaB and caspase-1. FEBS Lett.

[96] Case CL (2011). Regulating caspase-1 during Infection: roles of NLRs, AIM2, and ASC. Yale J Biol Med.

[97] Sollberger G, Strittmatter GE, Garstkiewicz M, Sand J, Beer HD (2014). Caspase-1: the inflammasome and beyond. Innate Immun.

[98] Barosova H, Karakocak BB, Septiadi D, Petri-Fink A, Stone V, Rothen-Rutishauser B (2020). An in Vitro Lung System to assess the Proinflammatory Hazard of Carbon Nanotube Aerosols. Int J Mol Sci.

[99] Braakhuis HM, He R, Vandebriel RJ, Gremmer ER, Zwart E, Vermeulen JP, et al. An air-liquid interface bronchial epithelial model for realistic, repeated Inhalation exposure to Airborne particles for toxicity testing. J Vis Exp. 2020;159. 10.3791/61210. 10.3791/61210 32478724

[100] Ramachandran H, Martins S, Kontarakis Z, Krutmann J, Rossi A (2021). Fast but not furious: a streamlined selection method for genome-edited cells. Life Sci Alliance.

[101] Kampfer AAM, Busch M, Buttner V, Bredeck G, Stahlmecke B, Hellack B (2021). Model complexity as determining factor for in Vitro Nanosafety studies: effects of Silver and Titanium Dioxide nanomaterials in Intestinal models. Small.

[102] Livak KJ, Schmittgen TD (2001). Analysis of relative gene expression data using real-time quantitative PCR and the ∆∆CT method. Methods.

[103] Busch M, Bredeck G, Kampfer AAM, Schins RPF (2021). Investigations of acute effects of polystyrene and polyvinyl chloride micro- and nanoplastics in an advanced in vitro triple culture model of the healthy and inflamed intestine. Environ Res.

