# Supplementary figures and images for: Saharan dust induces NLRP3-dependent inflammatory cytokines in an alveolar air-liquid interface co-culture model
Source: Part Fibre Toxicol. 2023 Oct 20;20:39. doi: 10.1186/s12989-023-00550-w (PMC10588053; doi:10.1186/s12989-023-00550-w)

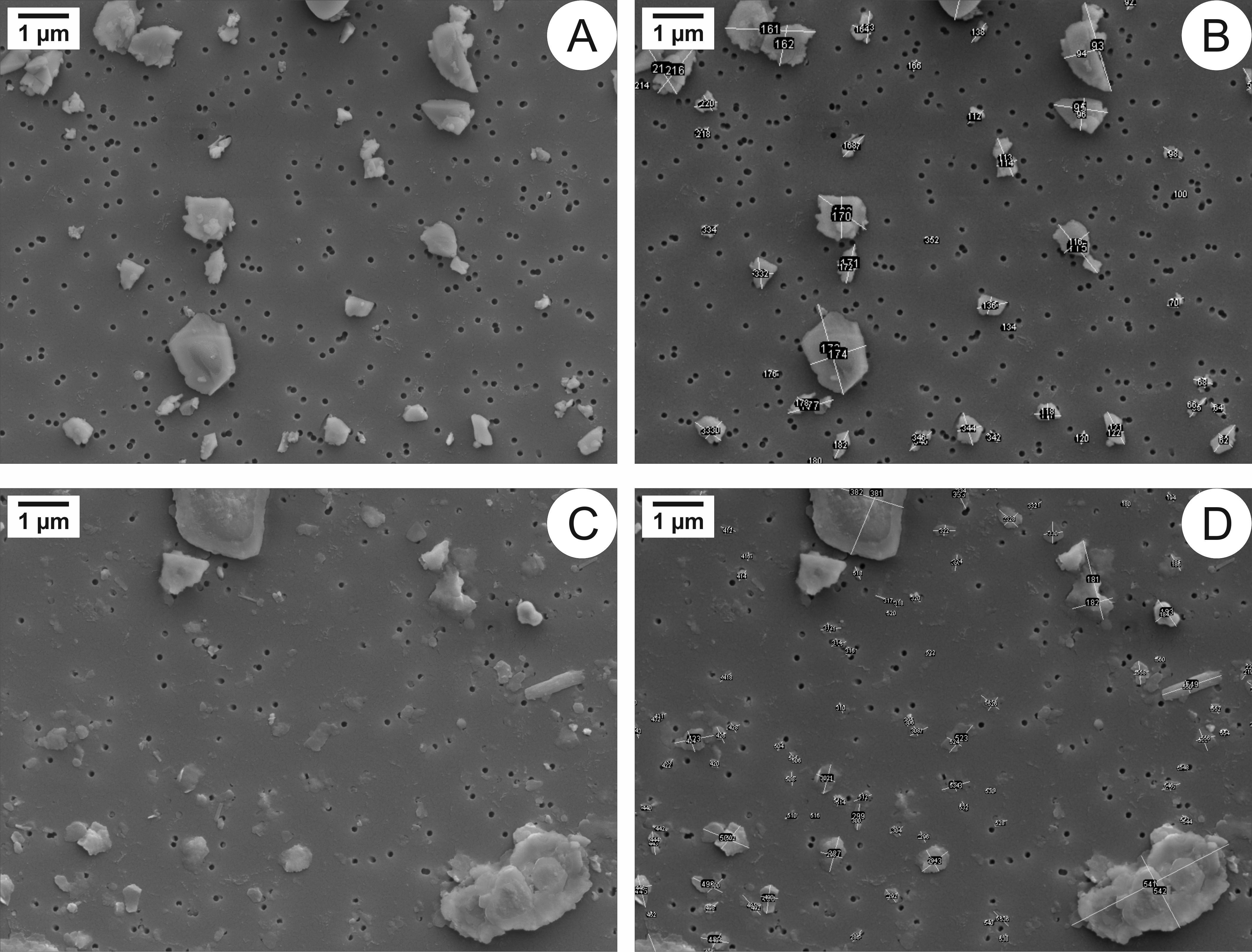

Supplement: Supplementary file 1 — Additional file 1: “Fig. S1.jpg”. Representative images of nebulized DQ12 and Saharan dust used for size determination. Suspensions of DQ12 quartz dust (A, B) and Saharan dust (C, D) in endotoxin-free H2O containing 1.25% PBS were sonicated and nebulized onto 0.1 μm pore-size nucleopore filters. Images were obtained at a nominal magnification of 5,000 x (pixel size: 6.2 nm). Images A and C show excerpts from images used for size determination. Images B and D show the same areas after size determination using ImageJ. [file 12989_2023_550_MOESM1_ESM.jpg]

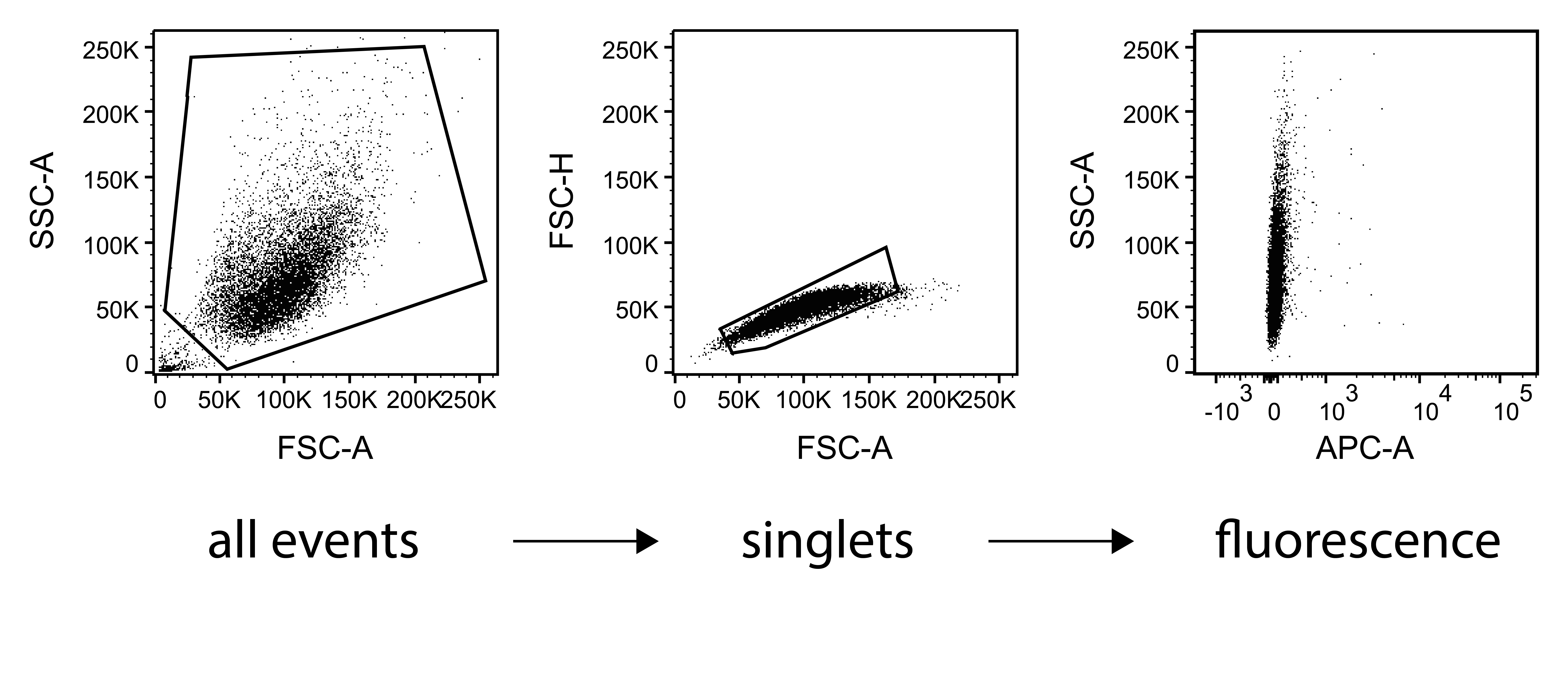

Supplement: Supplementary file 2 — Additional file 2: “Fig. S2.jpg”. Gating strategy of flow cytometry analysis. The first gate (SSC-A vs. FSC-A) was set to remove debris. Then doublets were discriminated (linear FSC-H vs. FSC-A) and median fluorescence intensity was determined in the APC-A channel. A representative dot plot is shown for differentiated unstained THP-1 wild type cells. The gating strategy is identical for all samples. SSC: side scatter, FSC: forward scatter, APC: allophycocyanin, A: area, H: height. [file 12989_2023_550_MOESM2_ESM.jpg]

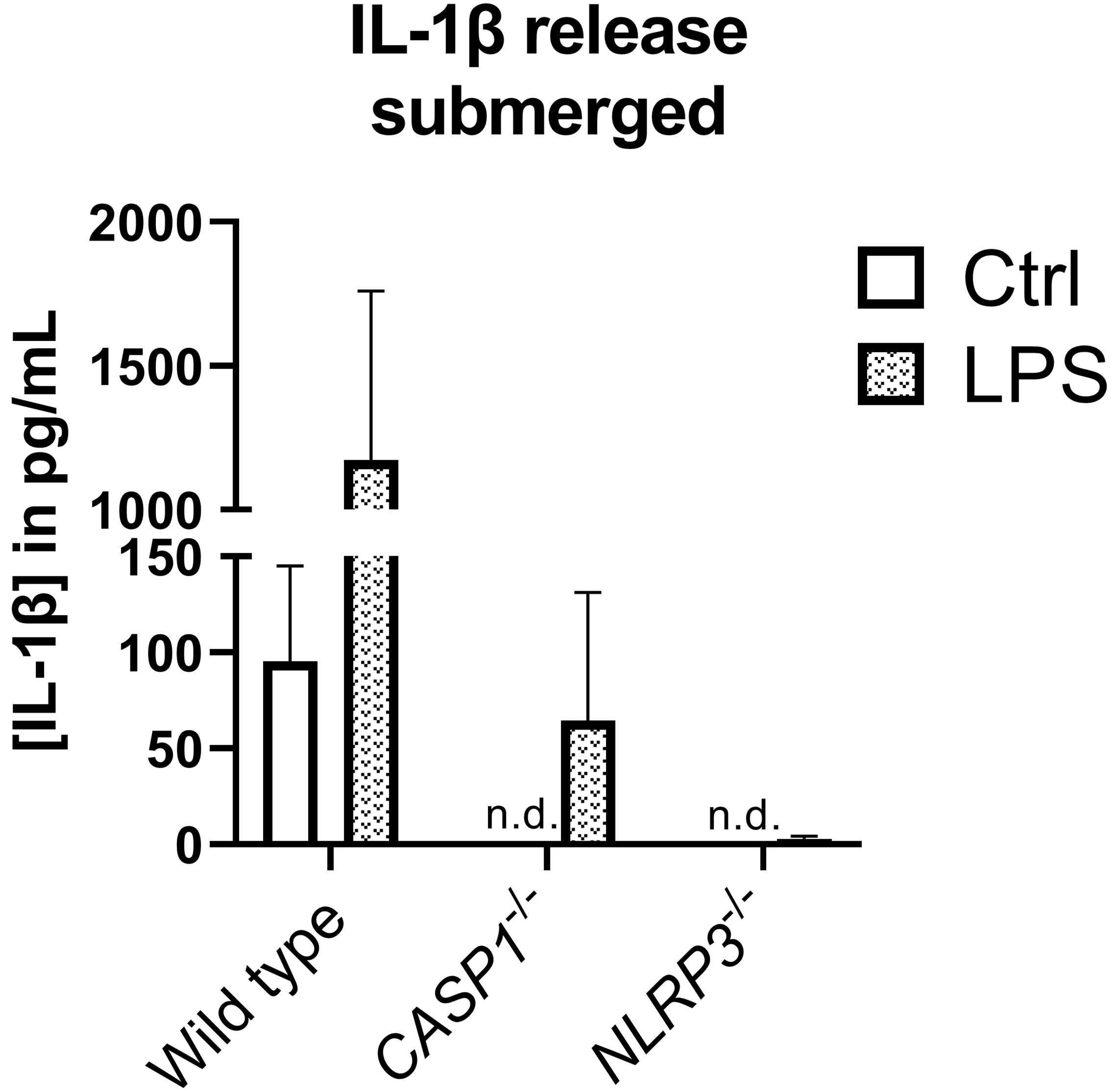

Supplement: Supplementary file 3 — Additional file 3: “Fig. S3.tif”. IL-1β release from THP-1 cells submerged. In parallel to ALI co-culture experiments (Figs. 3 and 7), the same wild type, CASP1−/−, and NLRP3−/− THP-1 cells were seeded in 24-well plates and exposed to 10 ng/mL LPS submerged for 24 h. In each experiment, one biological replicated was tested per group. The IL-1β concentrations in the supernatants were measured via ELISA. These submerged experiments were performed to confirm the genotypes of the THP-1 cells via comparison of the results to data from a previous study [55]. Depicted are means and standard deviations of N = 4 independent experiments. n.d.: not detected. [file 12989_2023_550_MOESM3_ESM.tif]

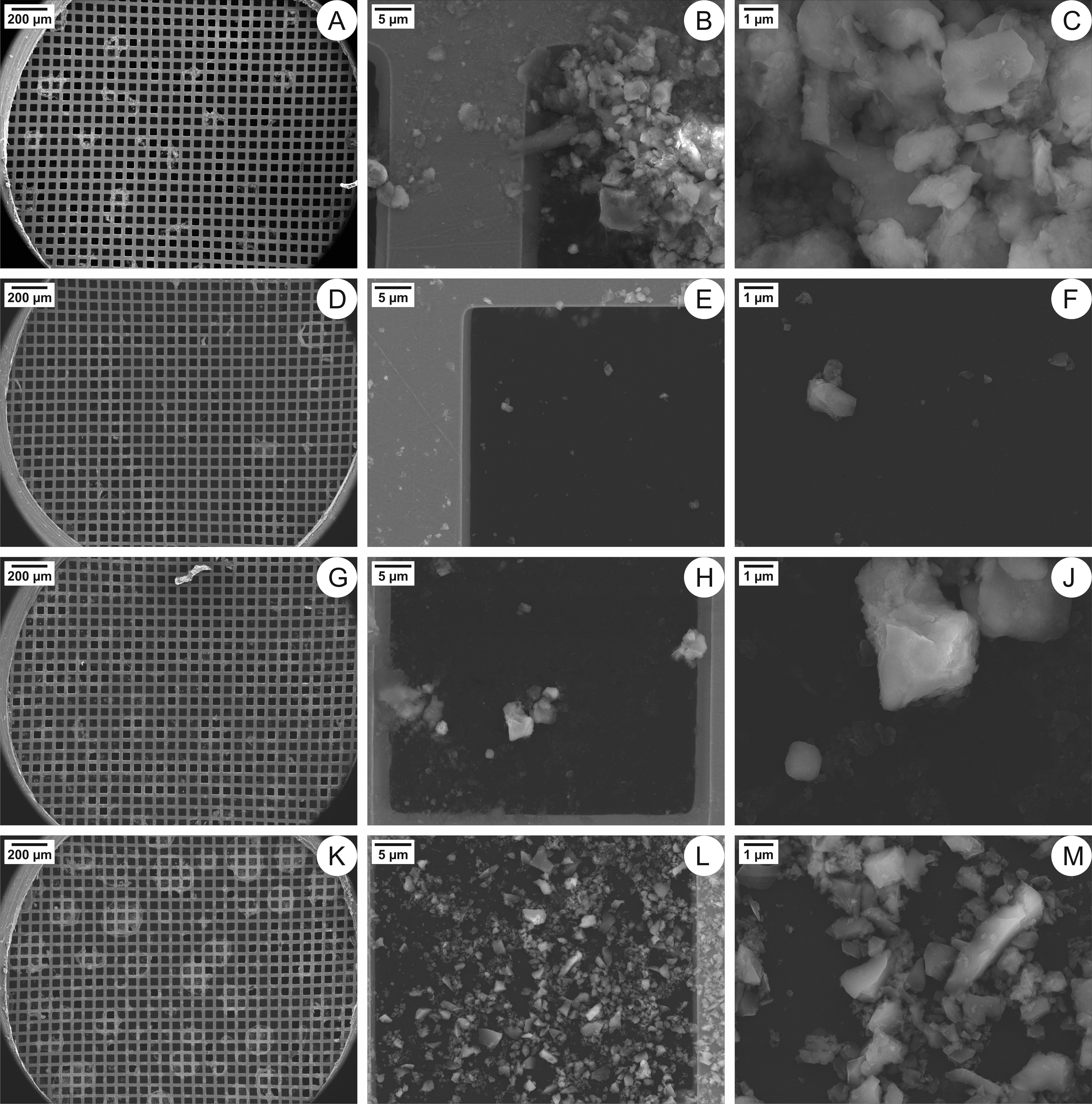

Supplement: Supplementary file 4 — Additional file 4: “Fig. S4.jpg”. Exemplary scanning electron microscopy images of Saharan dust and DQ12 quartz dust deposited on transmission electron microscopy grids during air-liquid interface exposure of co-cultures. A stainless steel insert with a TEM grid was placed into the Vitrocell Cloud 12α in parallel to ALI co-cultures and loaded with Saharan dust or DQ12, which were nebulized and deposited onto the inserts. Deposited doses of 10.9 µg/cm² SD (A-C), 10.4 µg/cm² DQ12 (D-F), 30.8 µg/cm² SD (G-J), and 30.1 µg/cm² DQ12 (K-M) were measured via quartz crystal microbalance. Images were obtained at nominal magnifications of 61 x (pixel size: 1.02 μm) (A, D, G, K), 2.5 kx (pixel size: 24.8 nm) (B, E, H, L), and 10 kx (pixel size: 6.2 nm) (C, F, J, M). [file 12989_2023_550_MOESM4_ESM.jpg]

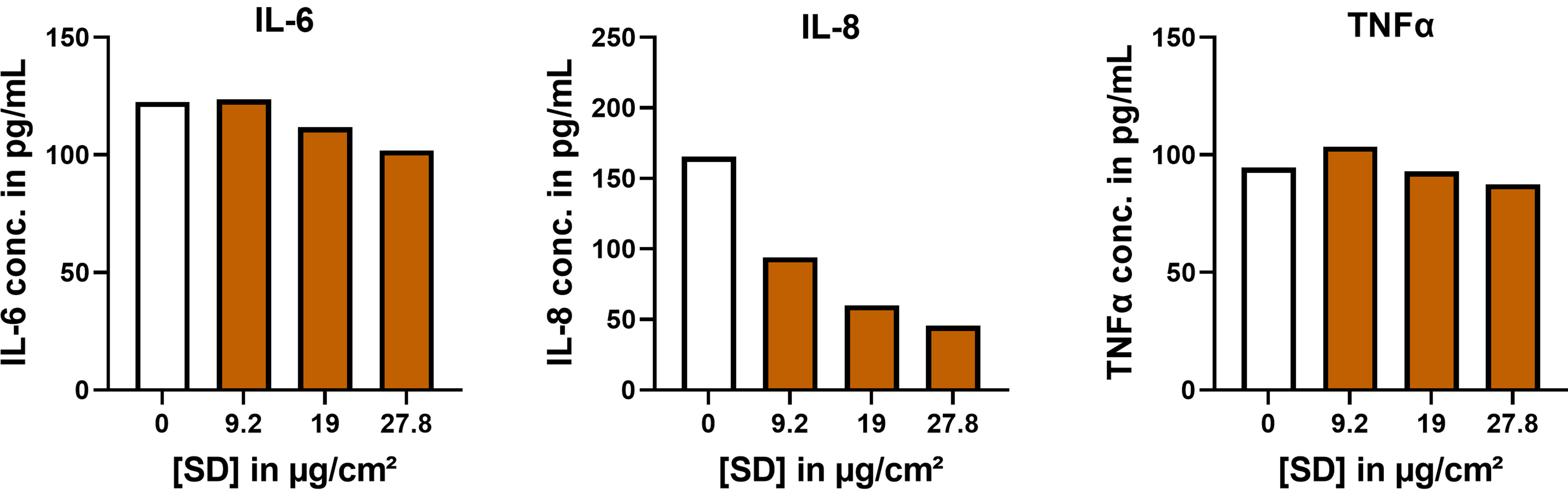

Supplement: Supplementary file 6 — Additional file 6: “Fig. S5.tif”. Interference with cytokine ELISAs. In a previous study, we found that SD but not DQ12 interfered with enzyme-linked immuno-sorbent assays (ELISAs) [21]. To assess the interaction of nebulized SD with IL-1β, IL-6, IL-8, and TNFα ELISAs, we nebulized and deposited SD on mixtures of recombinant cytokines (N = 1). The control was tested in triplicate, and each dust concentration in duplicates. Cytokine concentrations were determined after incubation for 24 h. SD weakly decreased the recovery of IL-6 by about 15% at the highest tested concentration of 27.8 µg/cm². SD concentrations of 9.2–27.8 µg/cm² decreased the recovery of IL-8 more strongly and dose-dependently by about 45–75%. SD barely affected the recovery of TNFα. For IL-1β, the interference could not be analyzed. Even in the negative control, IL-1β could not be detected after incubation in stainless steel inserts for 24 h. Considering the much lower interference of SD with IL-1β than with IL-8 in submerged experiments [21], at most a weak interference of SD with IL-1β is expected. In addition, in the interference experiment, SD was nebulized into a simulated apical compartment whereas basolateral supernatants were analyzed for cytokine release. Thus, even lower interferences in the ALI co-culture exposures than in this simulation are expected. [file 12989_2023_550_MOESM6_ESM.tif]

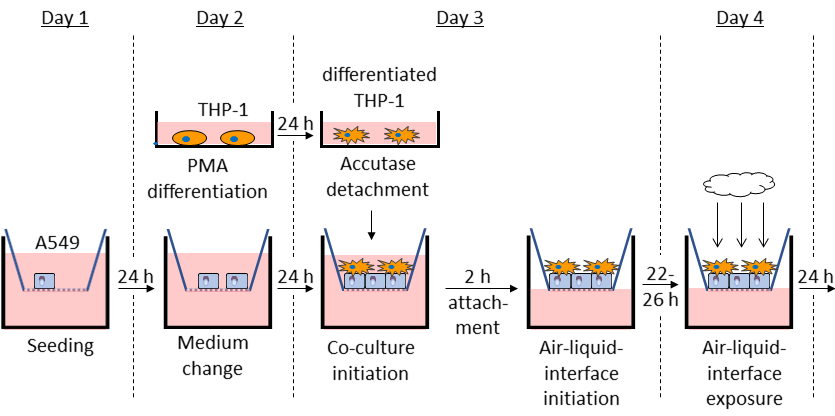

Supplement: Supplementary file 8 — Additional file 8: “Fig. S6.tif”. Air-liquid interface co-culture preparation. On day 1, A549 cells are seeded on the apical side of transwell inserts. On day 2, after incubation for 24 h, apical and basolateral medium are changed. Simultaneously, THP-1 cells are differentiated to macrophage-like cells through incubation with phorbol 12-myristate-13-acetate (PMA) for 24 h. On day 3, the differentiated THP-1 cells are detached with accutase and seeded on the apical side of the confluent A549 layer. Following attachment for 2 h, the apical medium is removed to initiate air-liquid interface (ALI) culture. On day 4, following 22–26 h of culture at the ALI, co-cultures are exposed at the ALI using a Vitrocell Cloud 12α. Co-cultures are exposed for 24 h. [file 12989_2023_550_MOESM8_ESM.tif]

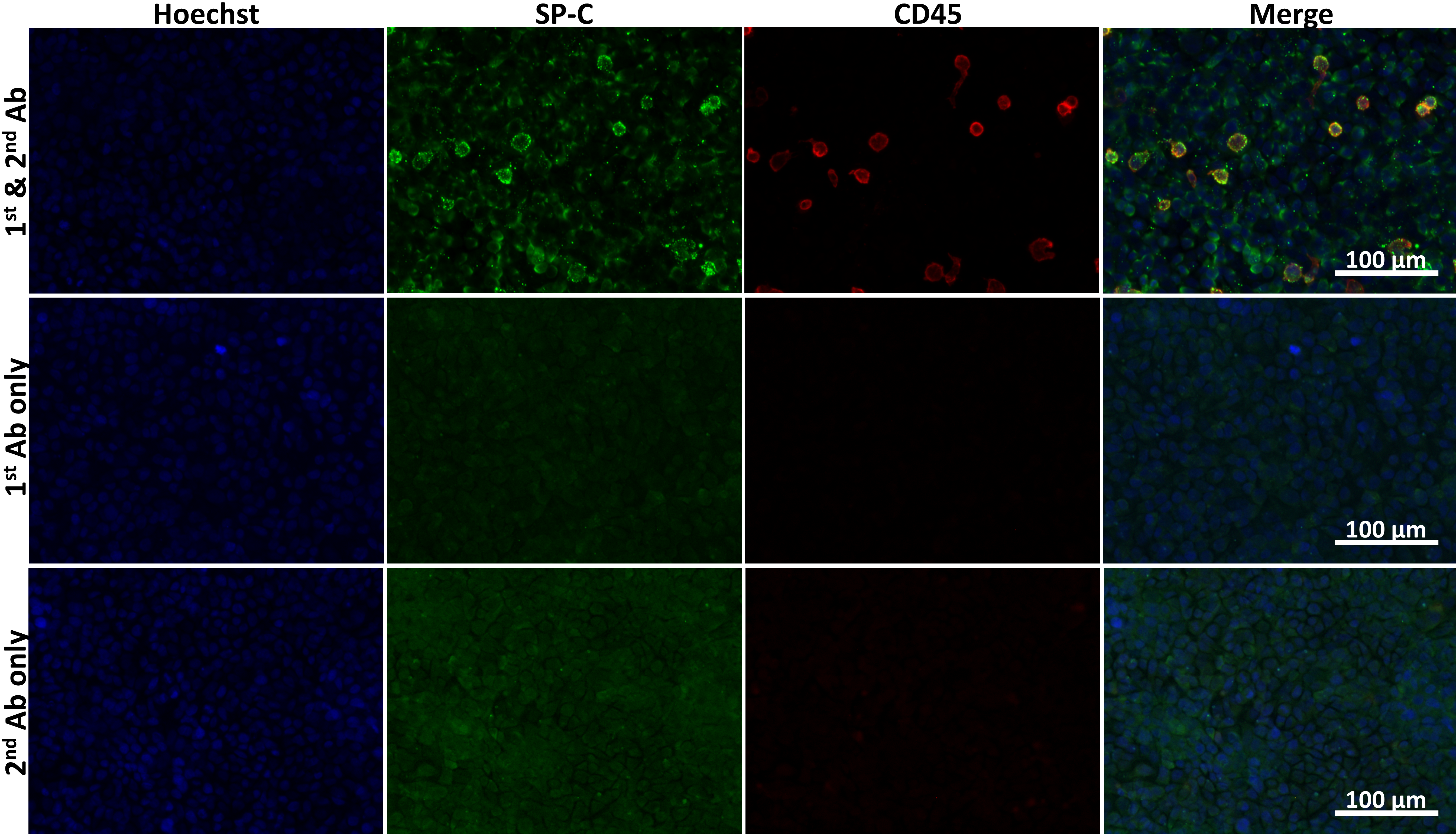

Supplement: Supplementary file 10 — Additional file 10: “Fig. S7.tif”. Control staining for surfactant protein C (SP-C) and CD45. Co-cultures of A549 cells with wild type THP-1 cells were fixed after 48 h of cultivation at the ALI. Nuclei were stained with Hoechst 33342. Immunostaining of SP-C and CD45 was performed using either primary and secondary antibodies, primary antibodies only, or secondary antibodies only. Representative images were obtained at 100 x magnification. [file 12989_2023_550_MOESM10_ESM.tif]

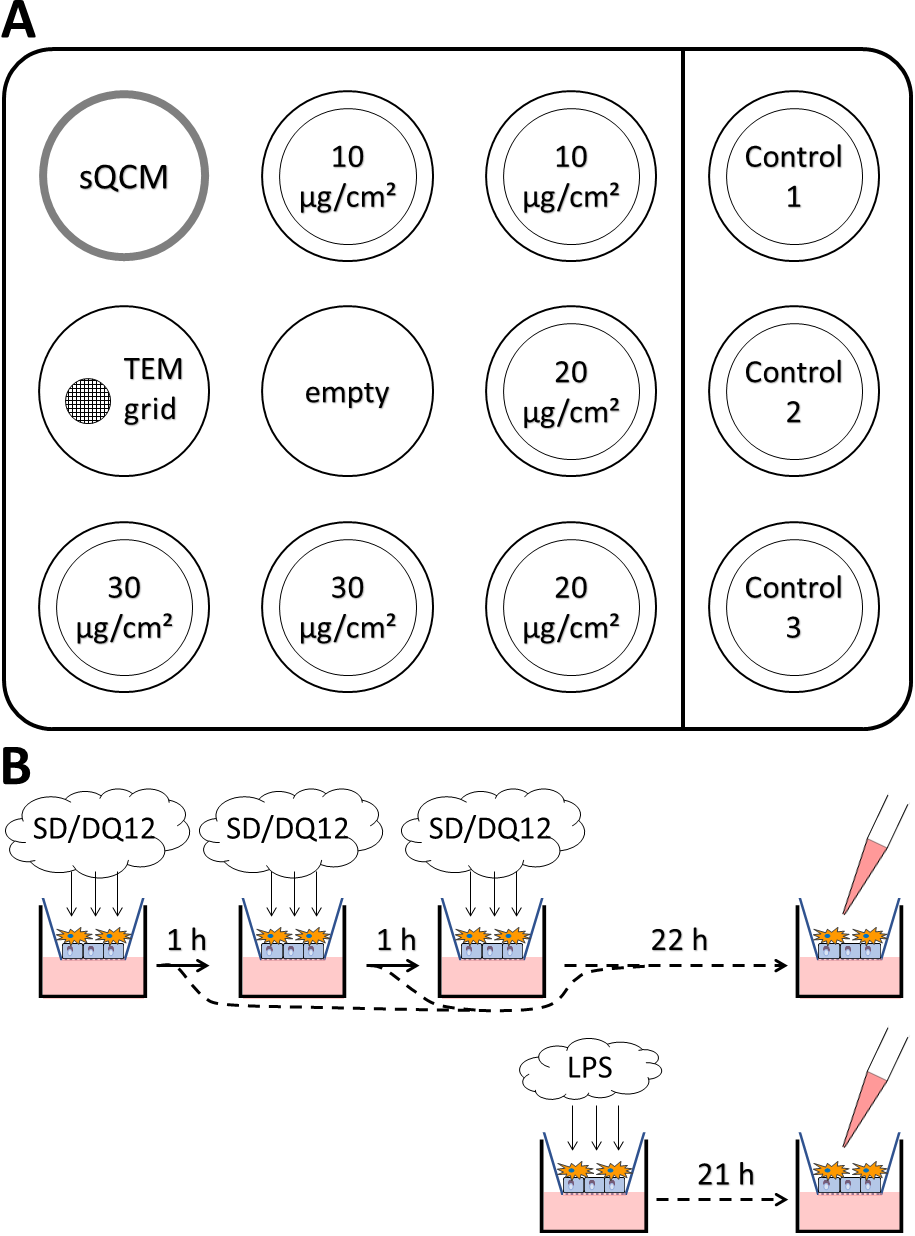

Supplement: Supplementary file 11 — Additional file 11: “Fig. S8.tif”. Exemplary loading pattern and procedure of Vitrocell Cloud 12α experiments. A: The bigger exposure chamber consists of nine wells and the smaller control chamber of three wells. The Vitrocell quartz crystal microbalance (sQCM) was installed in the top-left well. The middle well and the left-middle well of the exposure chamber were filled with 3 mL PBS. Optionally, a stainless-steel insert with a transmission electron microscopy (TEM) grid was placed into the middle-left position. The other wells were filled with 3 mL A549 medium containing 25 mM HEPES and co-culture inserts were placed on top. For each dose, one corner and one middle well were used. B: After each nebulization of SD or DQ12, sedimentation took about 30 min. The aerosol chamber was dismounted and optionally co-cultures could be removed from the Cloud system to obtain different doses. Subsequently, the aerosol chamber was mounted again and humidity was allowed to saturate for 30 min. This procedure was repeated twice. Directly after the third sedimentation of DQ12 and removing exposed co-cultures, the aerosol chamber and required wells for exposure to lipopolysaccharide (LPS) were cleaned and loaded. LPS was nebulized. All co-cultures were incubated until 24 h after the first nebulization of particles had passed. [file 12989_2023_550_MOESM11_ESM.tif]
