# Supplementary material for: Saharan dust induces NLRP3-dependent inflammatory cytokines in an alveolar air-liquid interface co-culture model
Source: Part Fibre Toxicol. 2023 Oct 20;20:39. doi: 10.1186/s12989-023-00550-w (PMC10588053; doi:10.1186/s12989-023-00550-w)
Supplement: Supplementary file 5 — Additional file 5: “Table S1.docx”. Depositions of DQ12, LPS, and SD in µg/cm² measured with Vitrocell sQCM. (belonging to Figs. 4, 5, and 6). Depositions from single experiments are presented with their means and standard deviations (St. dev.). For DQ12 and SD, accumulative doses after first (1x), second (2x), and third (3x) nebulization are shown. [file 12989_2023_550_MOESM5_ESM.docx]

|  | 1x DQ12 | 2x DQ12 | 3x DQ12 | LPS | 1x SD | 2x SD | 3x SD |
| --- | --- | --- | --- | --- | --- | --- | --- |
| Exp. 1 | 10.4 | 21.3 | 31.1 | 0.25 | 10.9 | 21.8 | 32.4 |
| Exp. 2 | 10.9 | 21.2 | 31.1 | 0.33 | 9.94 | 20.0 | 30.1 |
| Exp. 3 | 11.0 | 20.3 | 30.1 | 0.16 | 10.2 | 20.4 | 30.8 |
| Exp. 4 | 10.6 | 20.6 | 30.5 | 0.27 | 10.0 | 19.7 | 29.8 |
| Mean | 10.7 | 20.9 | 30.7 | 0.25 | 10.2 | 20.5 | 30.8 |
| St. dev. | 0.2 | 0.4 | 0.4 | 0.06 | 0.4 | 0.8 | 1.0 |
