# Supplementary material for: Saharan dust induces NLRP3-dependent inflammatory cytokines in an alveolar air-liquid interface co-culture model
Source: Part Fibre Toxicol. 2023 Oct 20;20:39. doi: 10.1186/s12989-023-00550-w (PMC10588053; doi:10.1186/s12989-023-00550-w)
Supplement: Supplementary file 7 — Additional file 7: “Table S2.docx”. Depositions of SD and LPS in µg/cm² measured with Vitrocell sQCM (belonging to Fig. 7). Depositions from single experiments are presented with their means and standard deviations (St. dev.). For SD, the accumulative doses after the third (3x) nebulization are shown. [file 12989_2023_550_MOESM7_ESM.docx]

|  | 3x SD | LPS |
| --- | --- | --- |
| Exp. 1 | 30.8 | 0.26 |
| Exp. 2 | 30.7 | 0.29 |
| Exp. 3 | N/A^1^ | N/A^1^ |
| Exp. 4 | 30.9 | 0.22 |
| Mean | 30.8 | 0.26 |
| St. dev. | 0.1 | 0.03 |

^1^The deposition could not be assessed, because the sQCM signal was interrupted during the experiment.
