# Supplementary material for: Saharan dust induces NLRP3-dependent inflammatory cytokines in an alveolar air-liquid interface co-culture model
Source: Part Fibre Toxicol. 2023 Oct 20;20:39. doi: 10.1186/s12989-023-00550-w (PMC10588053; doi:10.1186/s12989-023-00550-w)
Supplement: Supplementary file 9 — Additional file 9: “Supplementary material 1.docx”. ImageJ macro codes. Codes used to perform flat field corrections (A-D), rolling ball background subtraction (C, D), and merging (D) for images depicted in Fig. 3 and Fig. S7. [file 12989_2023_550_MOESM9_ESM.docx]

**Material 1. ImageJ macro codes.** Codes used to perform flat field corrections (A-D), rolling ball background subtraction (C, D), and merging (D) for images depicted in Fig. 3 and Fig. S7.

A: Hoechst 33342 staining

run("Clear Results");

extension = "czi";

inputDirectory = getDirectory("Select a directory containing one or several ."+extension+" files.");

fileList = getFileList(inputDirectory);

for (i = 0; i < fileList.length; i++)

{

run("Bio-Formats Importer", "open=" + inputDirectory+fileList[3*(i)] + " color_mode=Default view=Hyperstack stack_order=XYCZT");

MyImage = substring(getTitle(), 0, lengthOf(getTitle())-13);

run("Bio-Formats Importer", "open=" + inputDirectory+fileList[3*(i)+1] + " color_mode=Default view=Hyperstack stack_order=XYCZT");

run("Bio-Formats Importer", "open=" + inputDirectory+"221227_Background_Hoechst.czi color_mode=Default view=Hyperstack stack_order=XYCZT"); //open Background for Flat field correction

run("Measure");

mean = getResult("Mean", "row=nResults-1");

//run actual flat field correction

run("Select None");

run("Select None");

run("Calculator Plus", "i1="+fileList[3*(i)+1]+" i2=221227_Background_Hoechst.czi operation=[Divide: i2 = (i1/i2) x k1 + k2] k1="+mean+" k2=0 create");

rename("HoechstFFcorr");

run("Set Scale...", "distance=1.5477 known=1 unit=micron");

run("RGB Color");

run("Make Composite");

run("Split Channels");

selectWindow("C3-HoechstFFcorr");

run("Scale Bar...", "width=100 height=100 thickness=10 font=30 color=White background=None location=[Lower Right] horizontal bold overlay");

saveAs("tiff", inputDirectory+MyImage+ "_Hoechst.tiff");

close("*");

}

B: Immunostaining for surfactant protein C (SP-C)

run("Clear Results");

extension = "czi";

inputDirectory = getDirectory("Select a directory containing one or several ."+extension+" files.");

fileList = getFileList(inputDirectory);

for (i = 0; i < fileList.length; i++)

{

run("Bio-Formats Importer", "open=" + inputDirectory+fileList[3*(i)] + " color_mode=Default view=Hyperstack stack_order=XYCZT");

MyImage = substring(getTitle(), 0, lengthOf(getTitle())-13);

run("Bio-Formats Importer", "open=" + inputDirectory+fileList[3*(i)+2] + " color_mode=Default view=Hyperstack stack_order=XYCZT");

run("Bio-Formats Importer", "open=" + inputDirectory+"221227_Background_SFTPC.czi color_mode=Default view=Hyperstack stack_order=XYCZT"); //open Background for Flat field correction

run("Measure");

mean = getResult("Mean", "row=nResults-1");

//run actual flat field correction

run("Select None");

run("Select None");

run("Calculator Plus", "i1="+fileList[3*(i)+2]+" i2=221227_Background_SFTPC.czi operation=[Divide: i2 = (i1/i2) x k1 + k2] k1="+mean+" k2=0 create");

rename("SFTPCFFcorr");

run("Set Scale...", "distance=1.5477 known=1 unit=micron");

run("RGB Color");

run("Make Composite");

run("Split Channels");

selectWindow("C2-SFTPCFFcorr");

run("Scale Bar...", "width=100 height=100 thickness=10 font=30 color=White background=None location=[Lower Right] horizontal bold overlay");

saveAs("tiff", inputDirectory+MyImage+ "_SFTPC.tiff");

close("*");

}

C: Immunostaining for CD45

run("Clear Results");

extension = "czi";

inputDirectory = getDirectory("Select a directory containing one or several ."+extension+" files.");

fileList = getFileList(inputDirectory);

for (i = 0; i < fileList.length; i++)

{

run("Bio-Formats Importer", "open=" + inputDirectory+fileList[3*(i)] + " color_mode=Default view=Hyperstack stack_order=XYCZT");

MyImage = substring(getTitle(), 0, lengthOf(getTitle())-13);

run("Bio-Formats Importer", "open=" + inputDirectory+"221227_Background_CD45.czi color_mode=Default view=Hyperstack stack_order=XYCZT"); //open Background for Flat field correction

run("Measure");

mean = getResult("Mean", "row=nResults-1");

//run actual flat field correction

run("Select None");

run("Select None");

run("Calculator Plus", "i1="+fileList[3*(i)]+" i2=221227_Background_CD45.czi operation=[Divide: i2 = (i1/i2) x k1 + k2] k1="+mean+" k2=0 create");

rename("CD45FFcorr");

//run rolling ball background subtraction

run("Subtract Background...", "rolling=40");

run("Set Scale...", "distance=1.5477 known=1 unit=micron");

run("RGB Color");

run("Make Composite");

run("Split Channels");

selectWindow("C1-CD45FFcorr");

run("Scale Bar...", "width=100 height=100 thickness=10 font=30 color=White background=None location=[Lower Right] horizontal bold overlay");

saveAs("tiff", inputDirectory+MyImage+ "_merge.tiff");

close("*");

}

D: Merged image

run("Clear Results");

extension = "czi";

inputDirectory = getDirectory("Select a directory containing one or several ."+extension+" files.");

fileList = getFileList(inputDirectory);

for (i = 0; i < fileList.length; i++)

{

run("Bio-Formats Importer", "open=" + inputDirectory+fileList[3*(i)] + " color_mode=Default view=Hyperstack stack_order=XYCZT");

MyImage = substring(getTitle(), 0, lengthOf(getTitle())-13);

run("Bio-Formats Importer", "open=" + inputDirectory+"221227_Background_CD45.czi color_mode=Default view=Hyperstack stack_order=XYCZT"); //open Background for Flat field correction

run("Measure");

mean = getResult("Mean", "row=nResults-1");

//run actual flat field correction

run("Select None");

run("Select None");

run("Calculator Plus", "i1="+fileList[3*(i)]+" i2=221227_Background_CD45.czi operation=[Divide: i2 = (i1/i2) x k1 + k2] k1="+mean+" k2=0 create");

rename("CD45FFcorr");

//run rolling ball background subtraction

run("Subtract Background...", "rolling=40");

run("Bio-Formats Importer", "open=" + inputDirectory+fileList[3*(i)+1] + " color_mode=Default view=Hyperstack stack_order=XYCZT");

run("Bio-Formats Importer", "open=" + inputDirectory+"221227_Background_Hoechst.czi color_mode=Default view=Hyperstack stack_order=XYCZT"); //open Background for Flat field correction

run("Measure");

mean = getResult("Mean", "row=nResults-1");

//run actual flat field correction

run("Select None");

run("Select None");

run("Calculator Plus", "i1="+fileList[3*(i)+1]+" i2=221227_Background_Hoechst.czi operation=[Divide: i2 = (i1/i2) x k1 + k2] k1="+mean+" k2=0 create");

rename("HoechstFFcorr");

run("Bio-Formats Importer", "open=" + inputDirectory+fileList[3*(i)+2] + " color_mode=Default view=Hyperstack stack_order=XYCZT");

run("Bio-Formats Importer", "open=" + inputDirectory+"221227_Background_SFTPC.czi color_mode=Default view=Hyperstack stack_order=XYCZT"); //open Background for Flat field correction

run("Measure");

mean = getResult("Mean", "row=nResults-1");

//run actual flat field correction

run("Select None");

run("Select None");

run("Calculator Plus", "i1="+fileList[3*(i)+2]+" i2=221227_Background_SFTPC.czi operation=[Divide: i2 = (i1/i2) x k1 + k2] k1="+mean+" k2=0 create");

rename("SFTPCFFcorr");

run("Merge Channels...", "c1=CD45FFcorr c2=SFTPCFFcorr c3=HoechstFFcorr create"); //1 red, 2 green, 3 blue

run("Set Scale...", "distance=1.5477 known=1 unit=micron");

run("RGB Color");

run("Scale Bar...", "width=100 height=100 thickness=10 font=30 color=White background=None location=[Lower Right] horizontal bold overlay");

saveAs("png", inputDirectory+MyImage+ "_merge.png"); //for scale bar

saveAs("tiff", inputDirectory+MyImage+ "_merge.tiff"); //for quality

close("*");

}
