# Supplementary material for: Saharan dust induces NLRP3-dependent inflammatory cytokines in an alveolar air-liquid interface co-culture model
Source: Part Fibre Toxicol. 2023 Oct 20;20:39. doi: 10.1186/s12989-023-00550-w (PMC10588053; doi:10.1186/s12989-023-00550-w)
Supplement: Supplementary file 12 — Additional file 12: “Table S3.docx”. qPCR primer pairs. Sequences and concentrations of the used qPCR primers and amplicon lengths and efficiencies of the used qPCR Primer pairs on both used devices. [file 12989_2023_550_MOESM12_ESM.docx]

| **Gene** |  | **Sequence (5’ 🡪 3’)** | **Working conc. (nM)** | | | **Amplicon length (bp)** | | | **Primer efficiency (%)** | | |  |
| --- | --- | --- | --- | --- | --- | --- | --- | --- | --- | --- | --- | --- |
|  |  |  | **MyiQ** | | **QS3** |  | **MyiQ** | | | **QS3** | |  |
| *ACTB* | fw | CCTGGCACCCAGCACAAT | 60 | 200 | | 70 | | 90.3 | | | 81.2 | |
|  | rv | GCCGATCCACACGGAGTACT | 60 | 200 | |  | |  | | |  | |
| *GAPDH* | fw | CCCCCACCACACTGAATCTC | 37.5 | 200 | | 65 | | 105.4 | | | 90.3 | |
|  | rv | GCCCCTCCCCTCTTCAAG | 37.5 | 200 | |  | |  | | |  | |
| *IL1B* | fw | GCCAGTGAAATGATGGCTTATT | 50 | 50 | | 82 | | 94.0 | | | 81.7 | |
|  | rv | AGGAGCACTTCATCTGTTTAGG | 50 | 50 | |  | |  | | |  | |
| *IL6* | fw | TCATCACTGGTCTTTTGGAG | 200 | 200 | | 161 | | 92.3 | | | 80.2 | |
|  | rv | GTCAGGGGTGGTTATTGC | 200 | 200 | |  | |  | | |  | |
| *IL8* | fw | ACTCCAAACCTTTCCACCC | 60 | 200 | | 168 | | 91.2 | | | 83.7 | |
|  | rv | CCCTCTTCAAAAACTTCTCCAC | 60 | 200 | |  | |  | | |  | |
| *TNFA* | fw | ACTTTGGAGTGATCGGCC | 200 | 200 | | 139 | | 102.4 | | | 94.4 | |
|  | rv | GCTTGAGGGTTTGCTACAAC | 200 | 200 | |  | |  | | |  | |
| *IL18* | fw | TCTTCATTGACCAAGGAAATCGG |  | 200 | | 75 | |  | | | 93.3 | |
|  | rv | TCCGGGGTGCATTATCTCTAC |  | 200 | |  | |  | | |  | |
| *HMOX1* | fw | ATGACACCAAGGACCAGAGCC | 200 | 200 | | 151 | | 91.6 | | | 86.9 | |
|  | rv | GTAAGGACCCATCGGAGAAGC | 200 | 200 | |  | |  | | |  | |
| *APE1/REF1* | fw | CTGCCTGGACTCTCTCATCAATAC | 200 | 200 | | 118 | | 92.3 | | | 94.6 | |
|  | rv | CCTCATCGCCTATGCCGTAAG | 200 | 200 | |  | |  | | |  | |
| *GGCS* | fw | TTGCAGGAAGGCATTGATCA | 200 | 200 | | 101 | | 103.0 | | | 89.3 | |
|  | rv | GCATCATCCAGGTGTATTTTCTCTT | 200 | 200 | |  | |  | | |  | |
| *NQO1* | fw | AACCACGAGCCCAGCCAAT |  | 200 | | 177 | |  | | | 96.3 | |
|  | rv | TGGCATAGAGGTCCGACTCC |  | 200 | |  | |  | | |  | |

MyiQ: MyiQ^TM^ cycler (Bio-Rad), QS3^TM^: QuantStudio 3 device (Thermo Fisher Scientific)
